# Supplementary material for: Structural basis for the regulation of human 5,10-methylenetetrahydrofolate reductase by phosphorylation and S-adenosylmethionine inhibition
Source: Nat Commun. 2018 Jun 11;9:2261. doi: 10.1038/s41467-018-04735-2 (PMC5995969; doi:10.1038/s41467-018-04735-2)
Supplement: Supplementary file 1 — Supplementary Information [file 41467_2018_4735_MOESM1_ESM.pdf]

Structural basis for the regulation of human 5,10-methylenetetrahydrofolate reductase by phosphorylation and S-adenosylmethionine inhibition

Froese et al.

1-MVNEARGNSSLNPCLEGSASSGSESSKDSSRCSTPGLDPERHERL-45

VNEARGNSSLNPCL

EARGNSSLNPCLEGSASSGSESSK

EARGNSSLNPCLEGSASSGSE

ARGNSSLNPCLEGSASSGS

SSLNPCLEGSASSGSESSKDSSR

LNPCLEGSASSGSESSKDSS

PCLEGSASSGSESSK

CLEGSASSGSESSKD

LEGSASSGSESSKDSSRCS

LEGSASSGSES

LEGSASSGSE

ASSGSESSKD

ASSGSESSK

TPGLDPERHERL

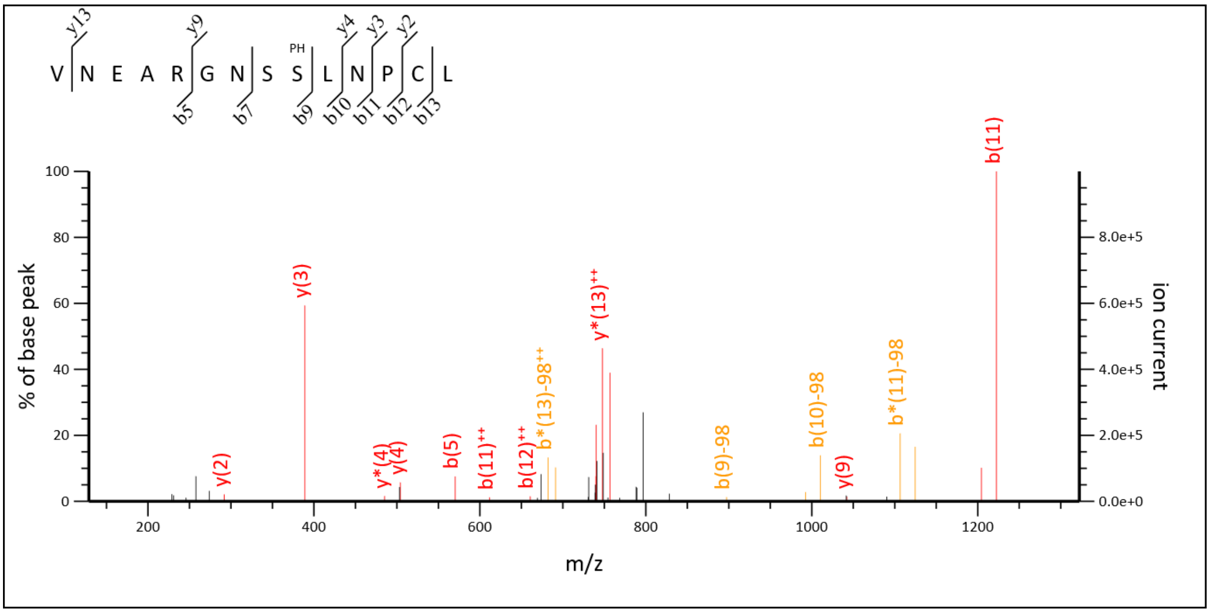

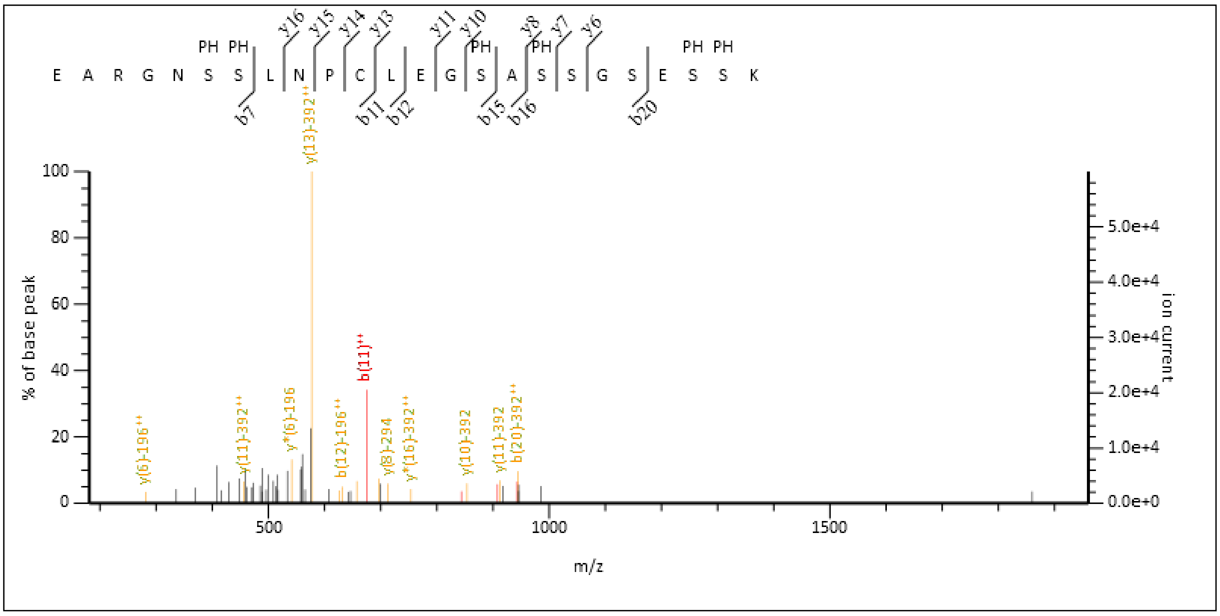

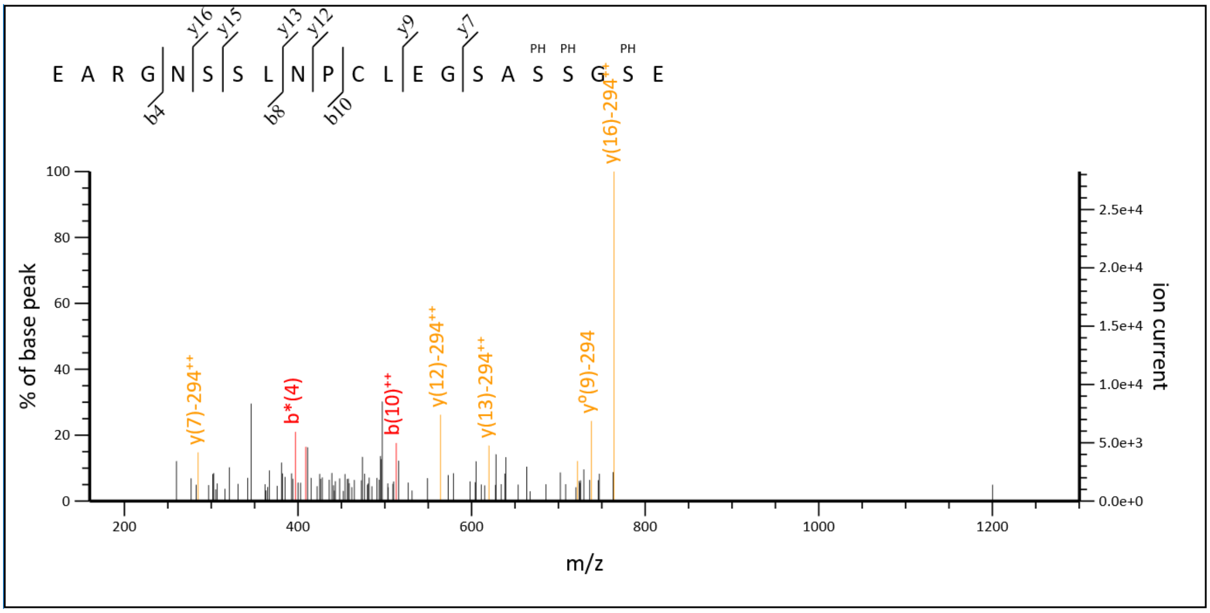
E A R G N S S L N P C L E G S A S S G S E
PH PH PH
y16 y15 y13 y12 y9 y7
b4 b8 b10
y(7)-294++
b*(4)
b(10)++
y(12)-294++
y(13)-294++
y°(9)-294
y(16)-294++
% of base peak
ion current
m/z
100
80
60
40
20
0
200
400
600
800
1000
1200
2.5e+4
2.0e+4
1.5e+4
1.0e+4
5.0e+3
0.0e+0

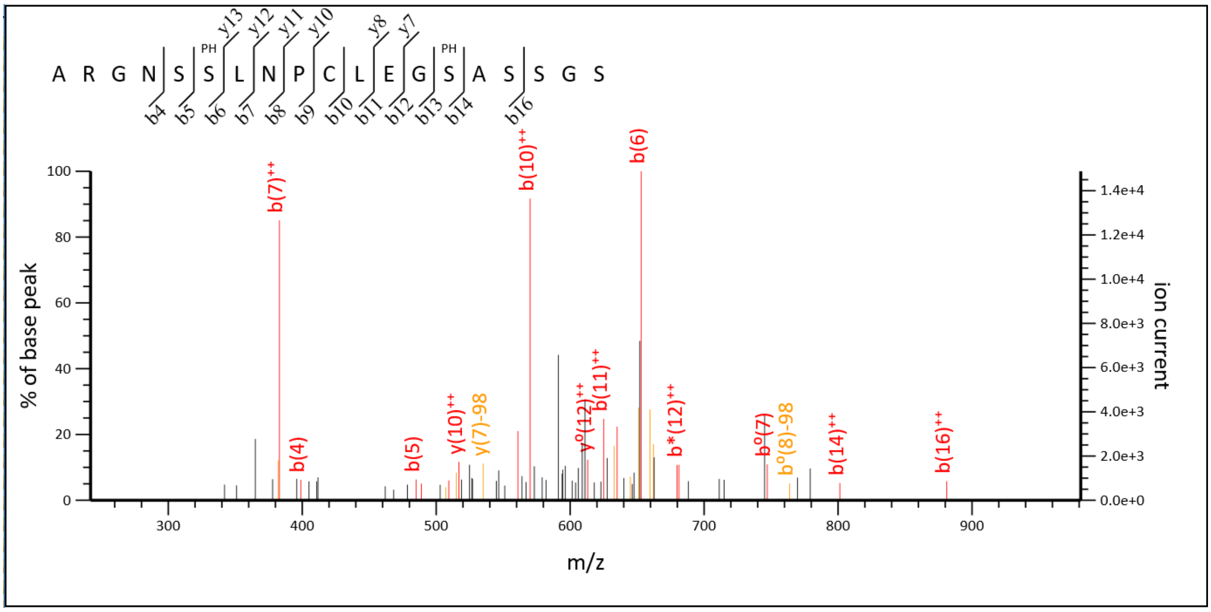
A R G N S S L N P C L E G S A S S G S
PH PH
y13 y12 y11 y10 y8 y7
b4 b5 b6 b7 b8 b9 b10 b11 b12 b13 b14 b16
b(7)++
b(4)
b(5)
y(10)++
y(7)-98
b(10)++
y°(12)++
b(11)++
b(6)
b*(12)++
b°(7)
b°(8)-98
b(14)++
b(16)++
% of base peak
ion current
m/z
100
80
60
40
20
0
300
400
500
600
700
800
900
1.4e+4
1.2e+4
1.0e+4
8.0e+3
6.0e+3
4.0e+3
2.0e+3
0.0e+0

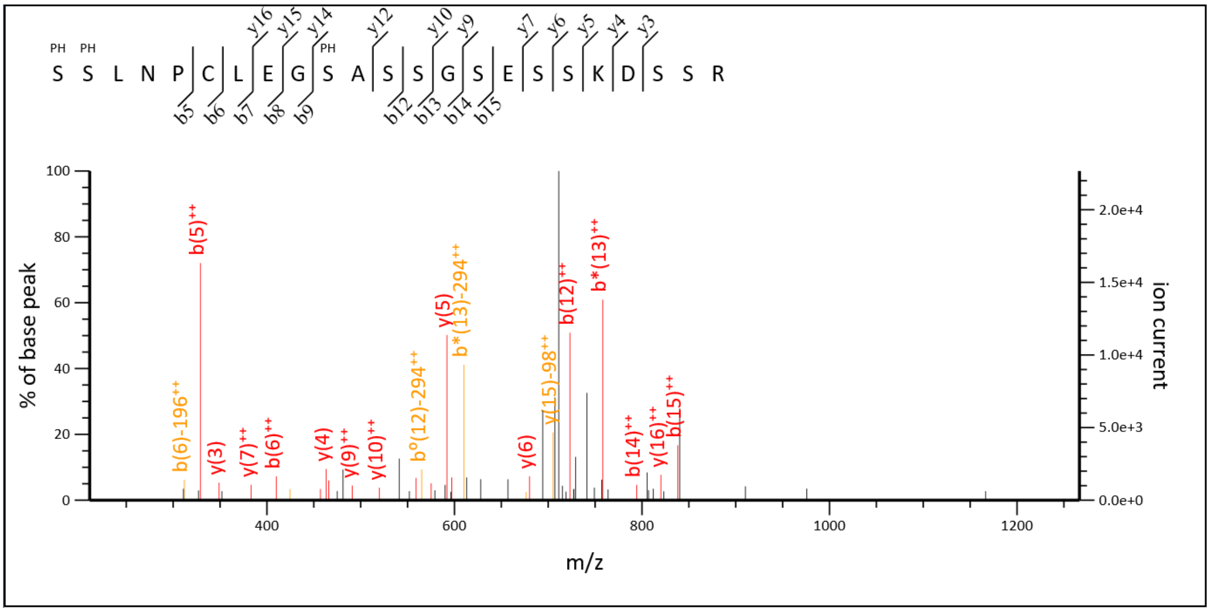
S S L N P C L E G S A S S G S E S S K D S S R
PH PH PH
y16 y15 y14 y12 y10 y9 y7 y6 y5 y4 y3
b5 b6 b7 b8 b9 b12 b13 b14 b15
b(6)-196++
b(5)++
y(3)
y(7)++
b(6)++
y(4)
y(9)++
y(10)++
b°(12)-294++
y(5)
b*(13)-294++
y(6)
y(15)-98++
b(12)++
b*(13)++
b(14)++
y(16)++
b(15)++
% of base peak
ion current
m/z
100
80
60
40
20
0
400
600
800
1000
1200
2.0e+4
1.5e+4
1.0e+4
5.0e+3
0.0e+0

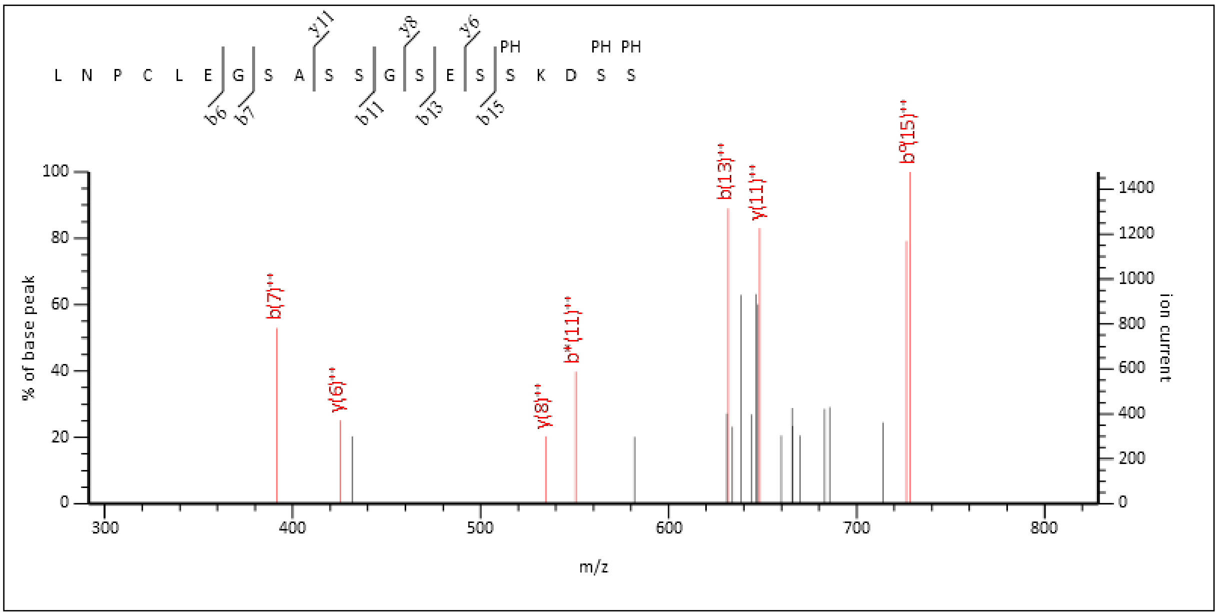
L N P C L E G S A S S G S E S S K D S S
PH PH PH
y11 y8 y6
b6 b7 b11 b13 b15
b(7)++
y(6)++
y(8)++
b*(11)++
b(13)++
y(11)++
b°(15)++
% of base peak
ion current
m/z

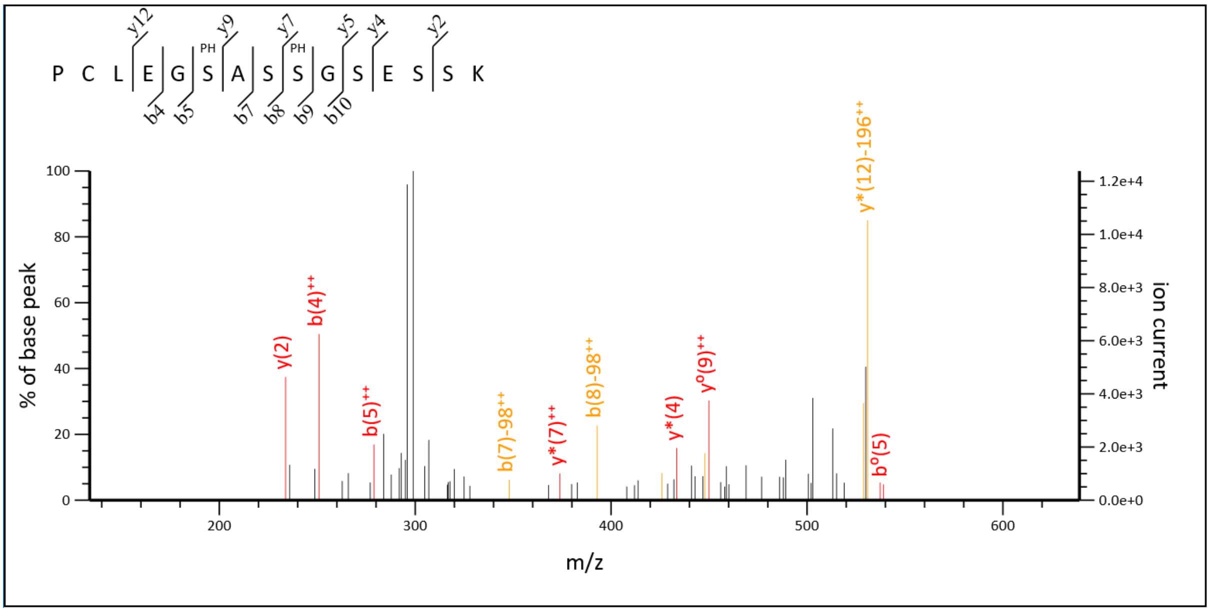
P C L E G S A S S G S E S S K
PH PH
y12 y9 y7 y5 y4 y2
b4 b5 b7 b8 b9 b10
y(2)
b(4)++
b(5)++
b(7)-98++
y*(7)++
b(8)-98++
y*(4)
y°(9)++
y*(12)-196++
b°(5)
% of base peak
ion current
m/z

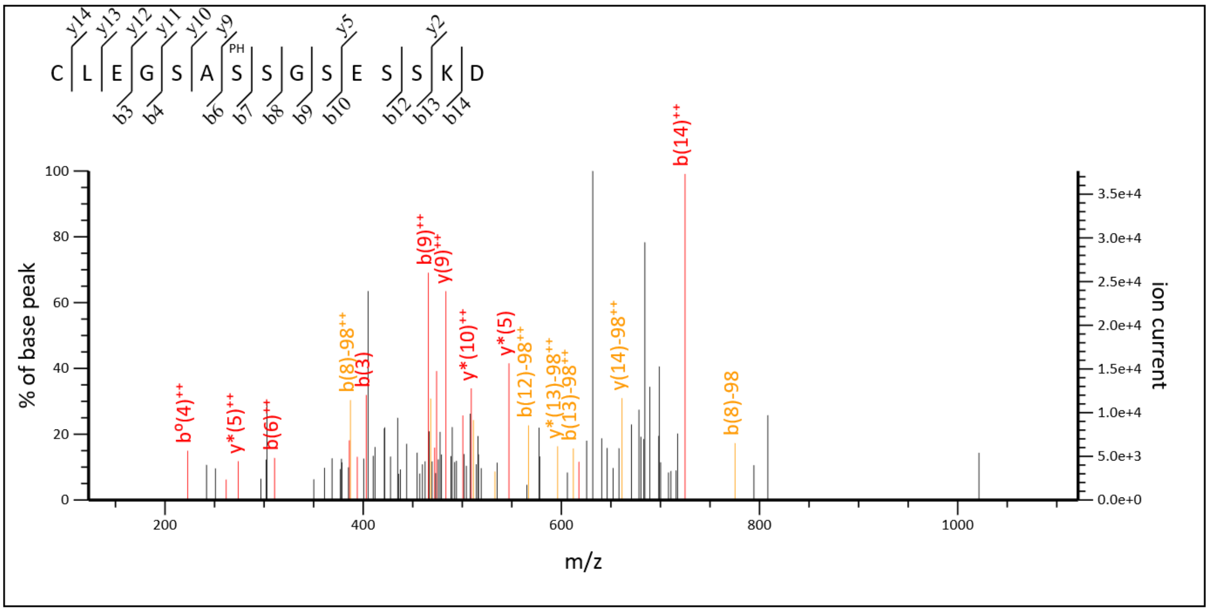
C L E G S A S S G S E S S K D
PH
y14 y13 y12 y11 y10 y9 y5 y2
b3 b4 b6 b7 b8 b9 b10 b12 b13 b14
b°(4)++
y*(5)++
b(6)++
b(8)-98++
b(3)
b(9)++
y(9)++
y*(10)++
y*(5)
b(12)-98++
y*(13)-98++
b(13)-98++
y(14)-98++
b(14)++
b(8)-98
% of base peak
ion current
m/z

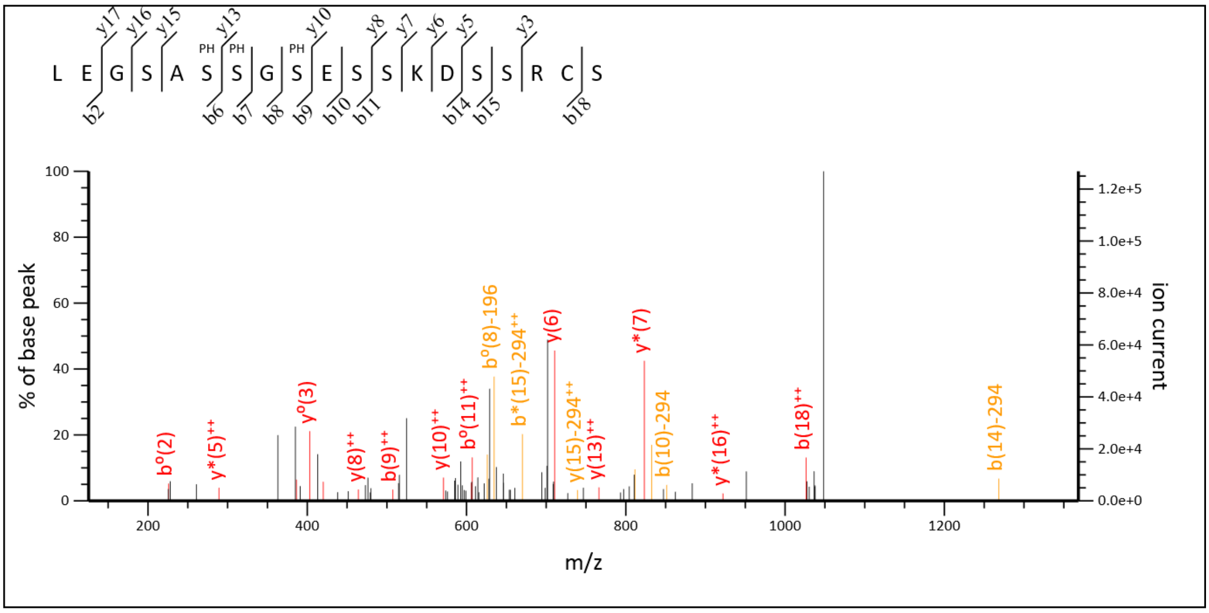

L E G S A S S G S E S S K D S S R C S
PH PH PH
y17 y16 y15 y13 y10 y8 y7 y6 y5 y3
b2 b6 b7 b8 b9 b10 b11 b14 b15 b18
% of base peak
ion current
m/z
bº(2)
y*(5)++
yº(3)
y(8)++
b(9)++
y(10)++
bº(11)++
bº(8)-196
b*(15)-294++
y(6)
y(15)-294++
y(13)++
y*(7)
b(10)-294
y*(16)++
b(18)++
b(14)-294

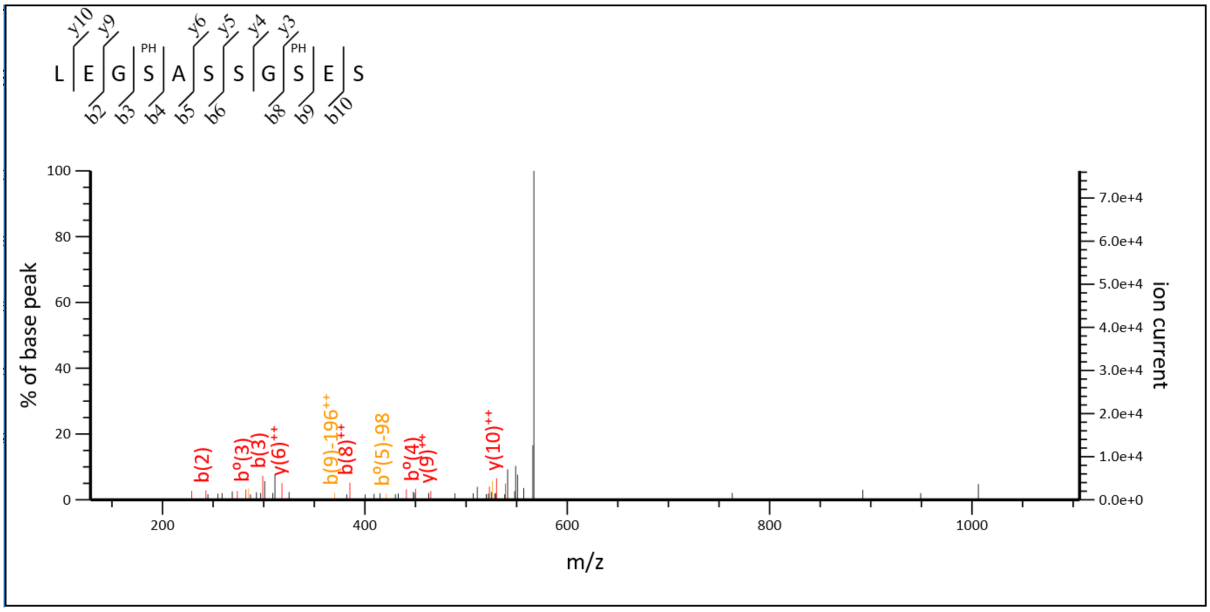

L E G S A S S G S E S
PH PH
y10 y9 y6 y5 y4 y3
b2 b3 b4 b5 b6 b8 b9 b10
% of base peak
ion current
m/z
b(2)
bº(3)
b(3)
y(6)++
b(9)-196++
b(8)++
bº(5)-98
bº(4)
y(9)++
y(10)++

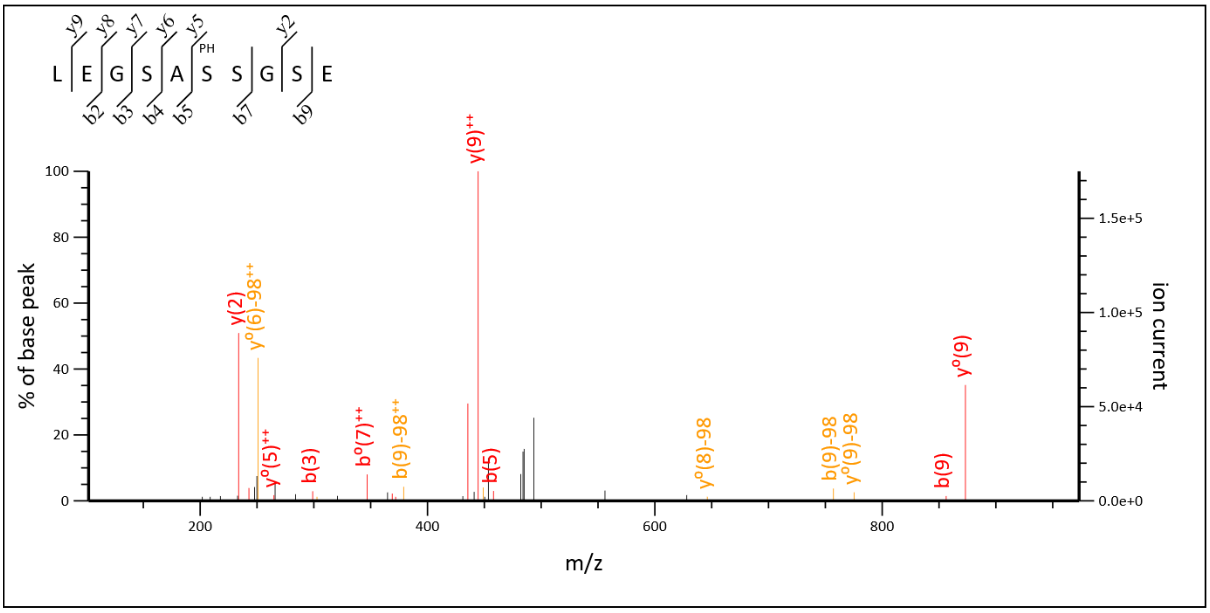

L E G S A S S G S E
PH
y9 y8 y7 y6 y5 y2
b2 b3 b4 b5 b7 b9
% of base peak
ion current
m/z
y(2)
yº(6)-98++
yº(5)++
b(3)
bº(7)++
b(9)-98++
y(9)++
b(5)
yº(8)-98
b(9)-98
yº(9)-98
b(9)
yº(9)

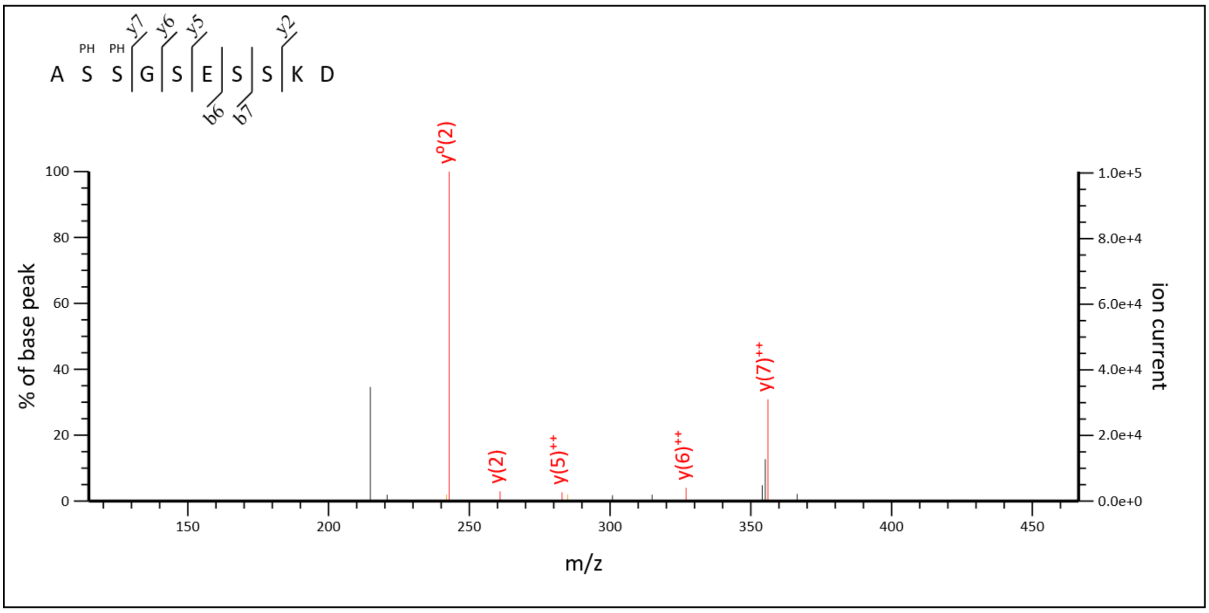
A S S G S E S S K D
PH PH
y7 y6 y5 y2
b6 b7
y°(2)
y(2)
y(5)++
y(6)++
y(7)++
% of base peak
ion current
m/z

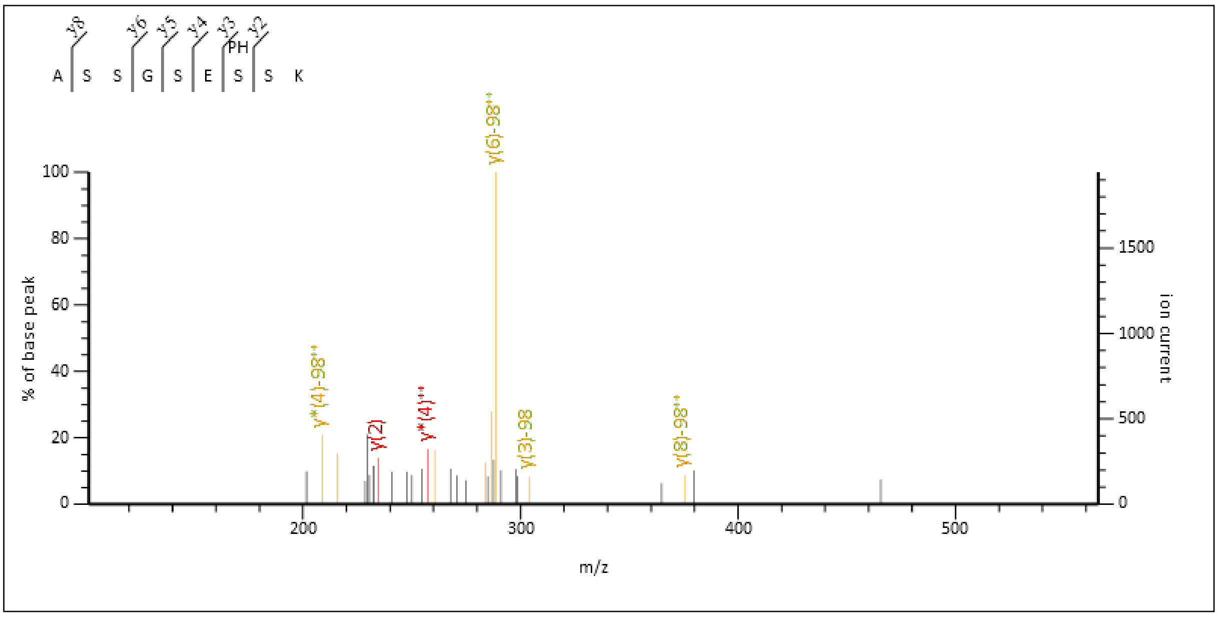
A S S G S E S S K
PH
y8 y6 y5 y4 y3 y2
y(6)-98++
y*(4)-98++
y(2)
y*(4)++
y(3)-98
y(8)-98++
% of base peak
ion current
m/z

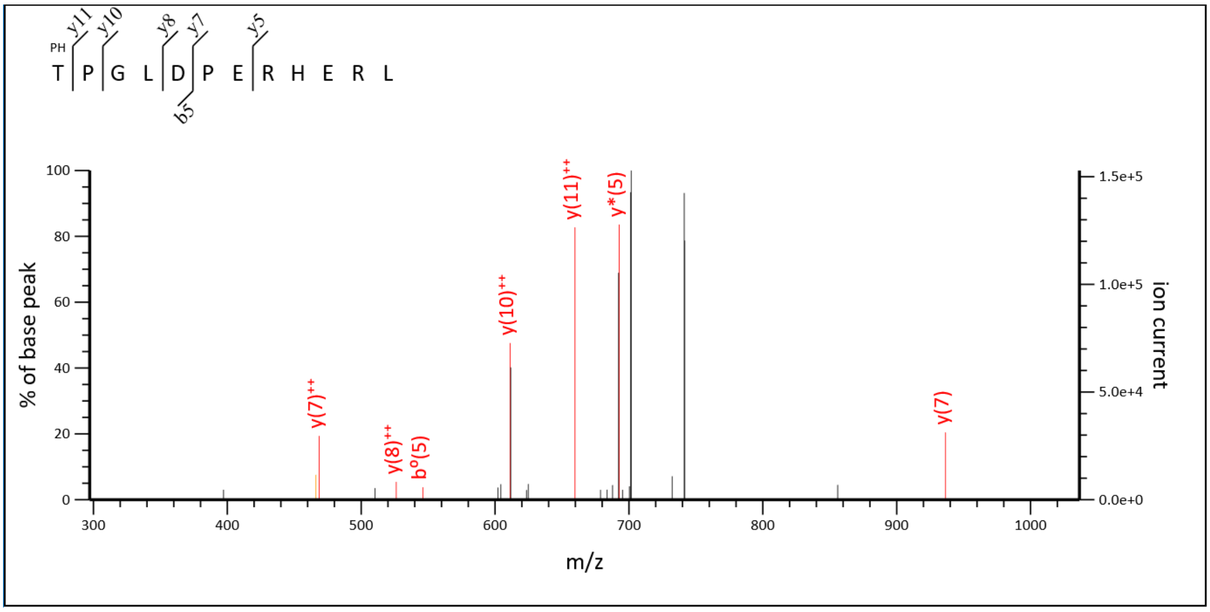
T P G L D P E R H E R L
PH
y11 y10 y8 y7 y5
b5
y(7)++
y(8)++
b°(5)
y(10)++
y(11)++
y*(5)
y(7)
% of base peak
ion current
m/z

87-GPLYIDVTWHPAGDPGSDKETSSM-110

GPLYIDVTWHPAGDPGSDKETSSM

TWHPAGDPGSDKE

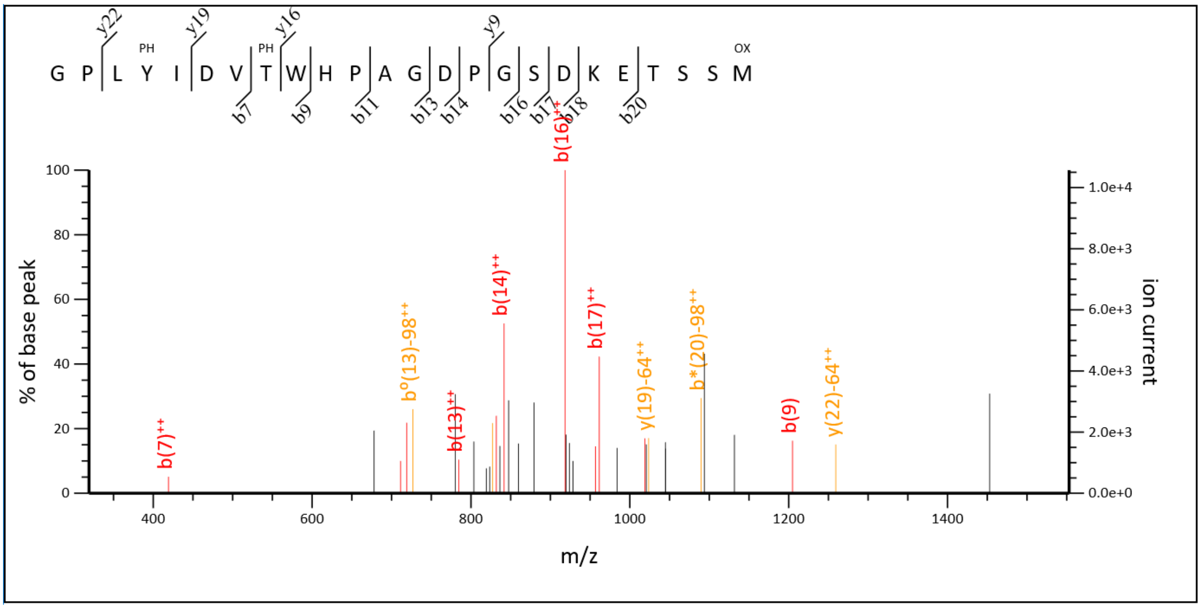

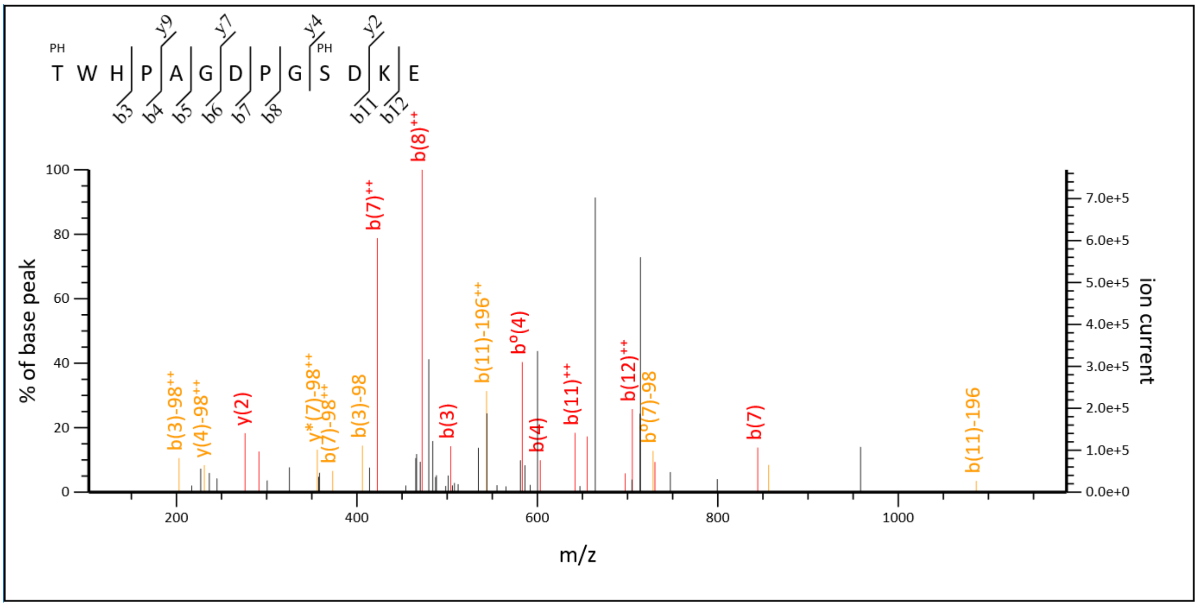

382-DEFPNGRWGNSSSPAF-397

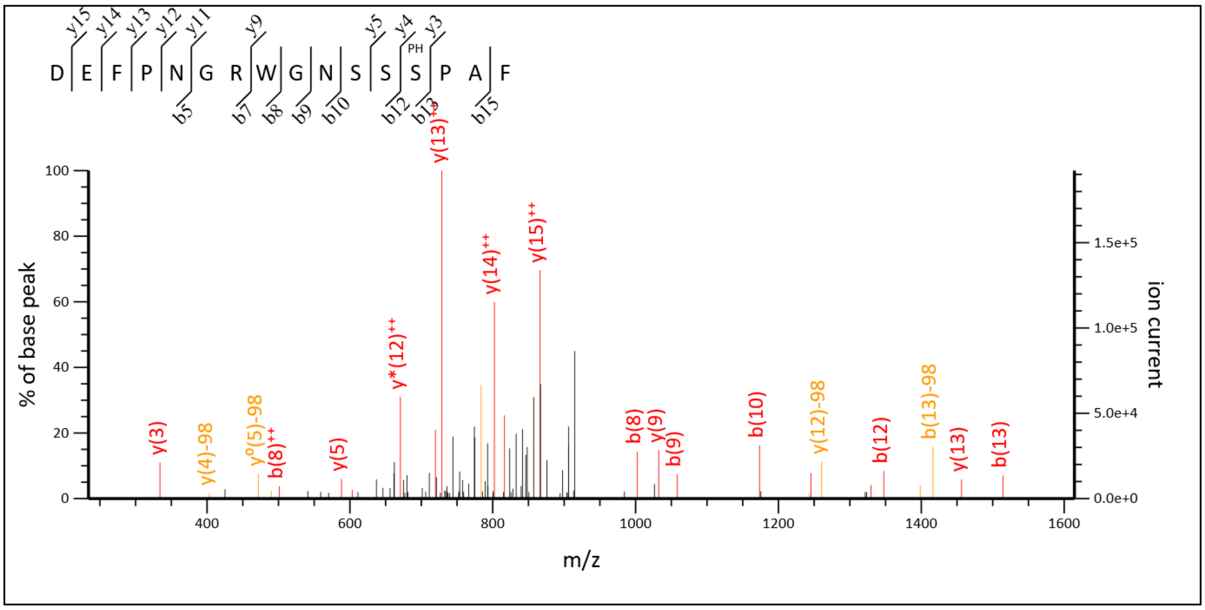

450-VTCLPWNDEPLAAETSLLK-468

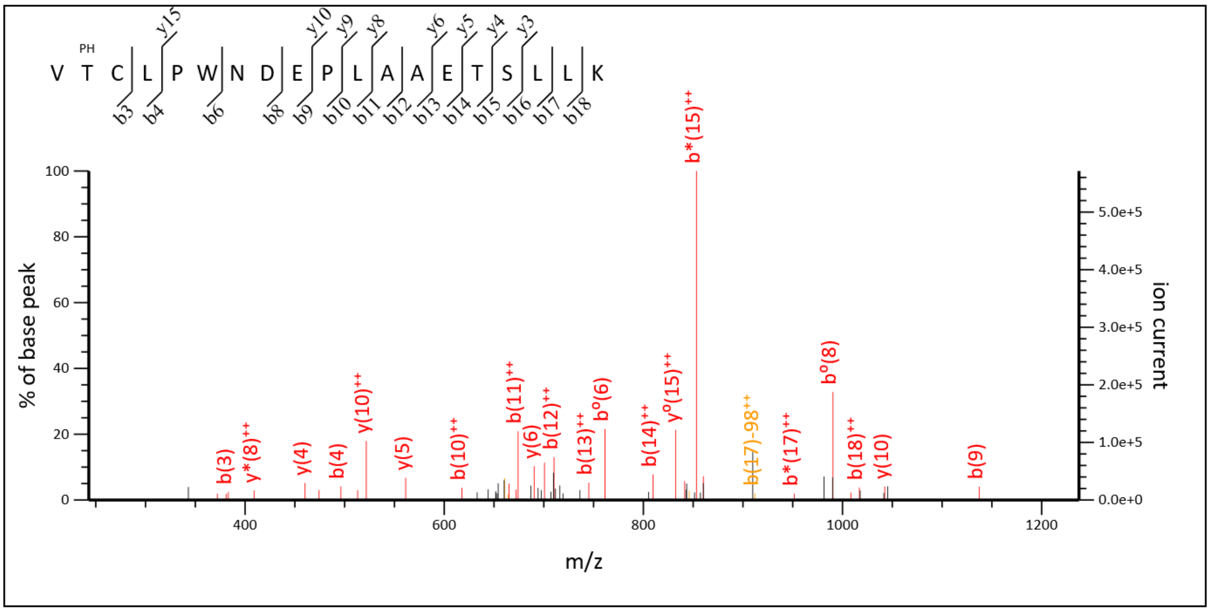

Supplementary Figure 1. Phosphomapping of *Hs*MTHFR$_{1\text{-}656}$. Annotated fragmentation spectra for 18 *Hs*MTHFR$_{1\text{-}656}$ phosphopetides from Mascot database searches including inset schematic showing peptide cleavages, b and y reporter ions and Mascot phosphosite assignments.

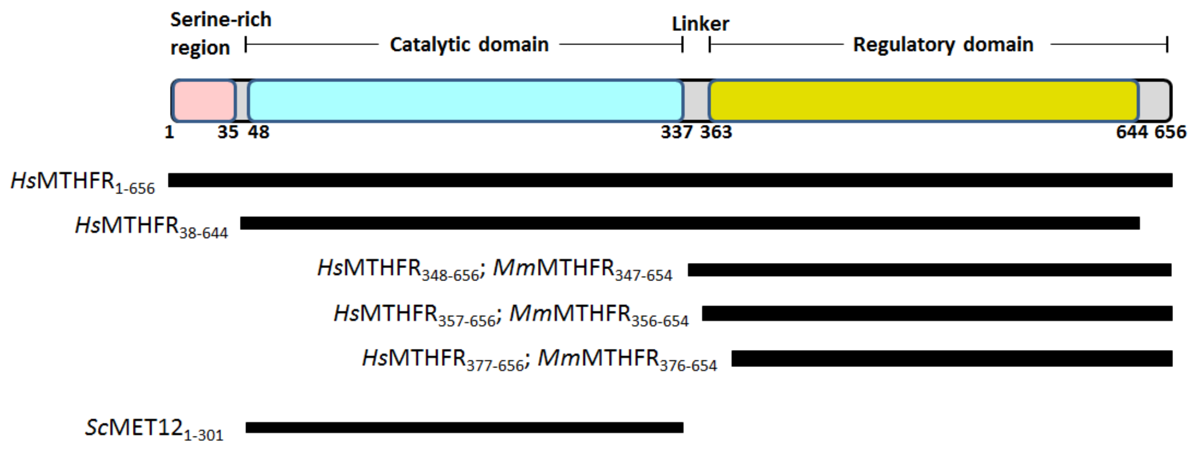

Supplementary Figure 2. Domain organization of MTHFR and constructs used. Domain organization of human MTHFR across evolution revealing differences in the N- and C-termini. Numbers given represent approximate amino acid boundaries in human MTHFR corresponding to NP_005948. Constructs with the prefix *Hs*MTHFR represent recombinant human protein, with the prefix *Mm*MTHFR represent recombinant mouse protein, and *Sc*MET12 represent the *Saccharomyces cerevisiae* MTHFR ortholog MET12.

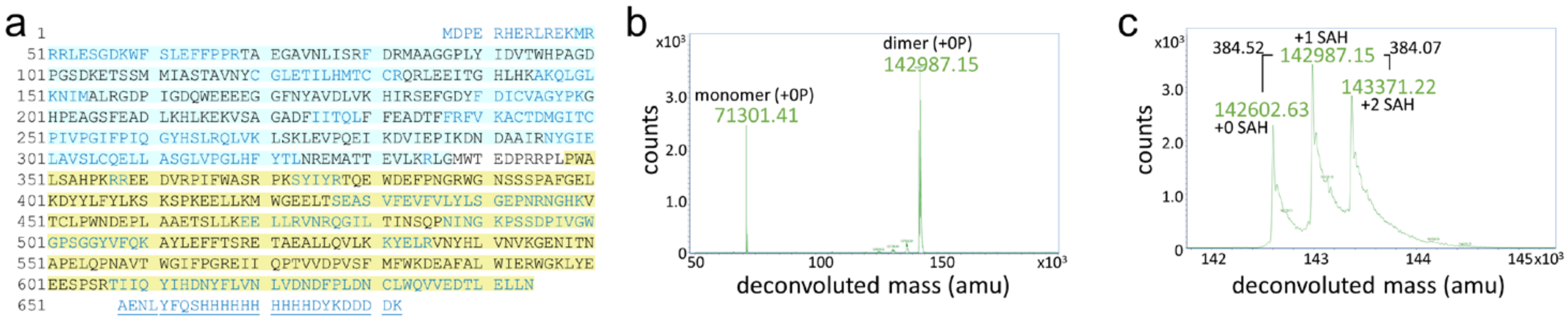

Supplementary Figure 3. Phosphorylation status of *Hs*MTHFR$_{38\text{-}644}$ and degradation of SAM to SAH following dephosphorylation. a Phosphorylation mapping of *Hs*MTHFR$_{38\text{-}644}$. The protein sequence is given as amino acids in single letter code, including the C-terminal His/flag-tag (underlined). Black font represents amino acids identified by the mass spectrometer (covered), blue font represents amino acids not identified (non-covered). Domains are coloured as in Figure 1. **b** Native mass spectrometry of as purified *Hs*MTHFR$_{38\text{-}644}$. Broad view including monomeric and dimeric forms of the protein. Monomer represents protein bound to 1 FAD (expected mass: 71299.56 amu); dimer represents protein bound to 2 FADs and 1 SAH (expected mass: 142983.34 amu). **c** Native mass spectrometry of as purified *Hs*MTHFR$_{38\text{-}644}$. Zoom in on peaks corresponding to sequential addition of SAH. Expected sizes: protein, 70513.90 amu; FAD, 785.56 amu; SAH, 384.42 amu. amu: atomic mass units

a

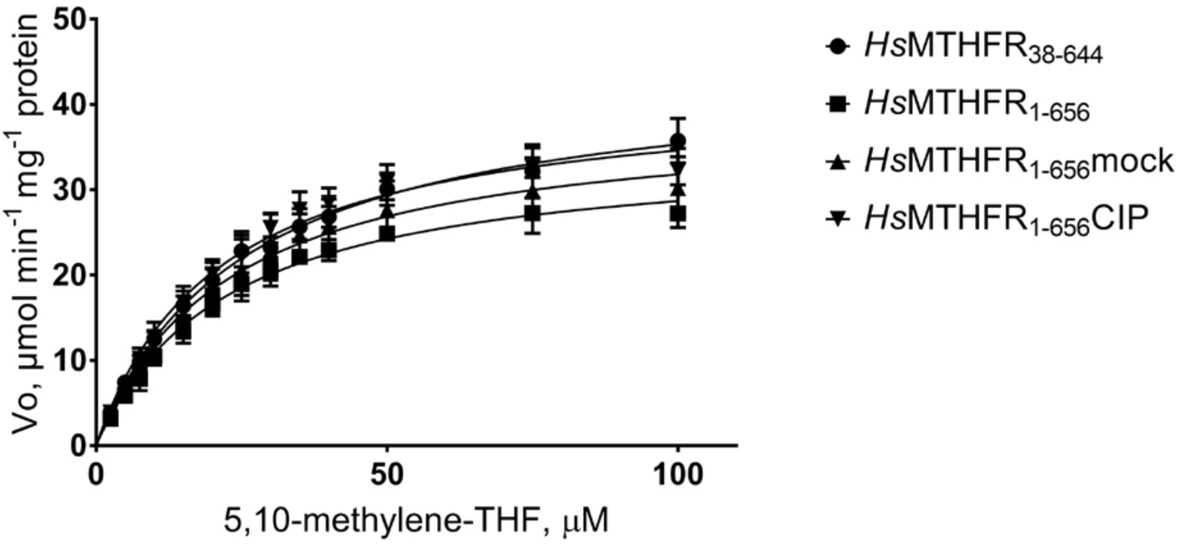

b

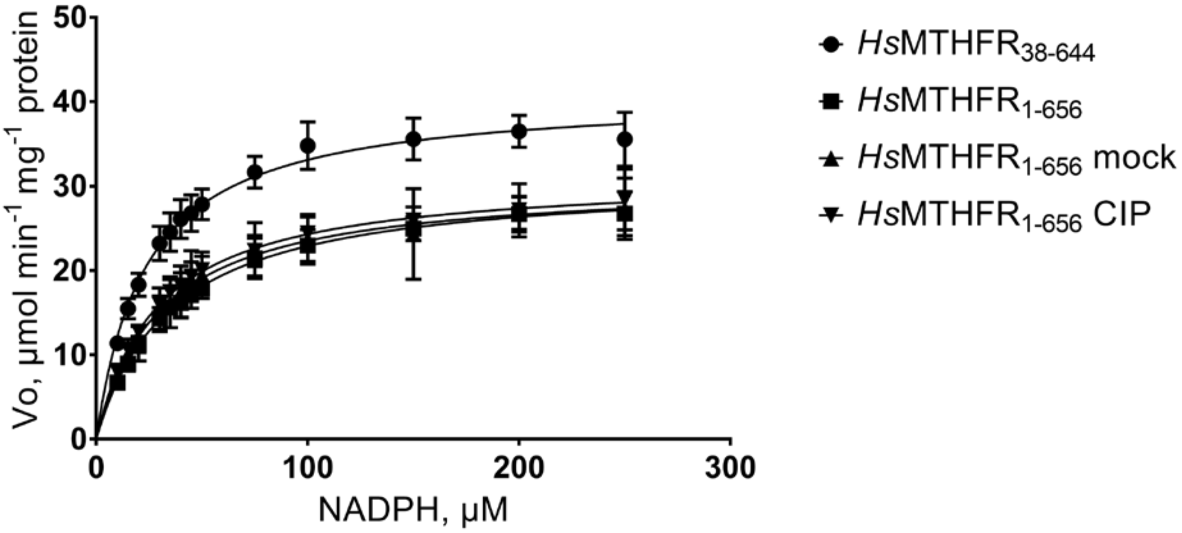

Supplementary Figure 4. Kinetic characterization of *Hs*MTHFR. a Michaelis-Menten kinetics of *Hs*MTHFR incubated with increasing concentrations of CH_2-THF. **b** Michaelis-Menten kinetics of *Hs*MTHFR incubated with increasing concentrations of NADPH. Both assays were performed as described in the Methods and summary data are provided in Table 1. Each value represents the results of at least 3 separate experiments and is given as ± S.D. Lines represent non-linear fit of Michaelis-Menten kinetics as calculated by GraphPad v.6.0.

a

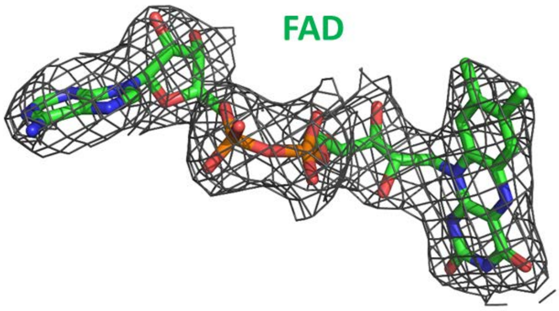

b

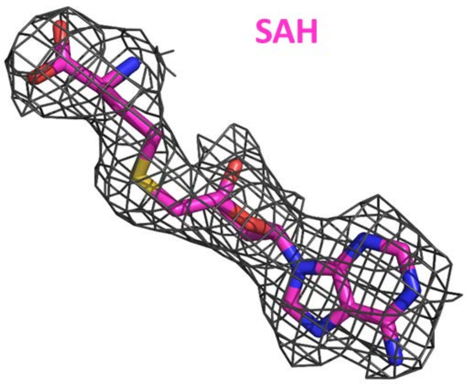

Supplementary Figure 5. Identification of bound ligands in the *Hs*MTHFR$_{38\text{-}644}$ crystal structure. **a** 2Fo-Fc, 1.5 SigmaA-weighted map of the electron density map for FAD in the catalytic domain. **b** 2Fo-Fc, 1.5 SigmaA-weighted map of the electron density map for SAH in the regulatory domain.

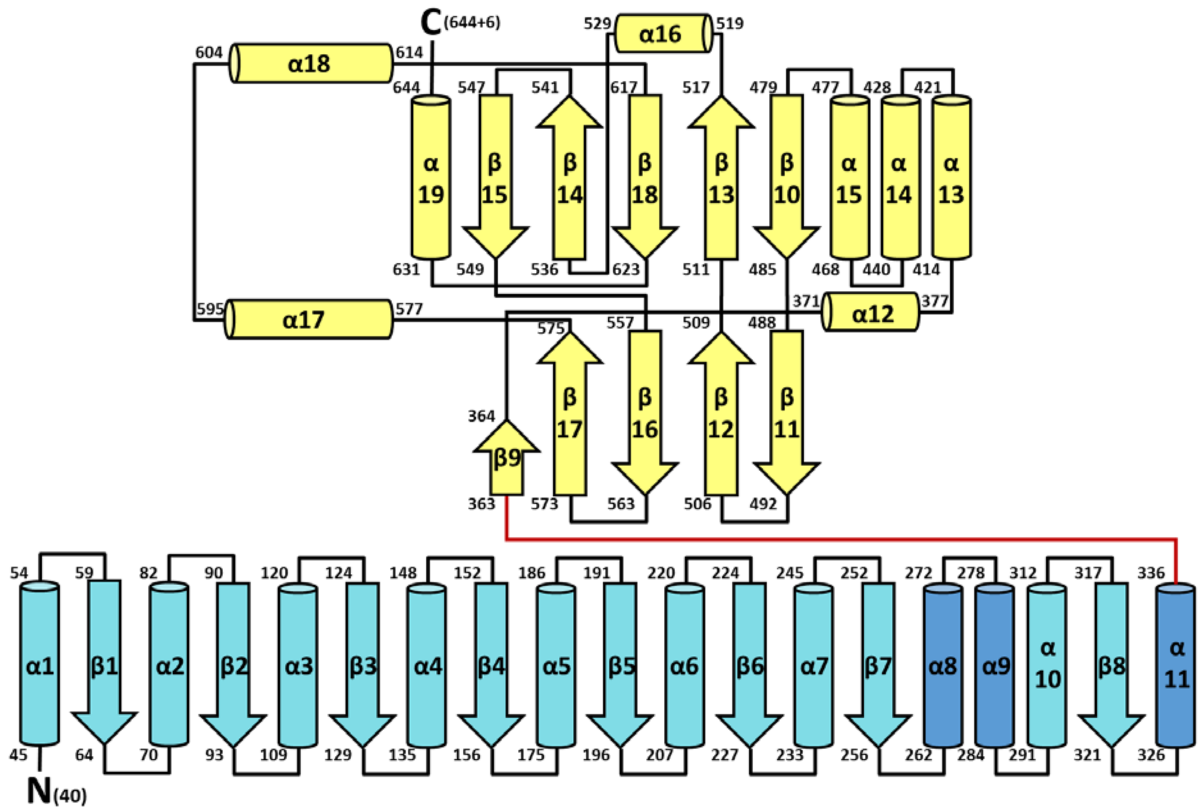

Supplementary Figure 6. Topology diagram of *Hs*MTHFR. Secondary structure is shown with the same colouring and labeling as in Figure 4, with the catalytic domain in cyan, the linker in red, and the regulatory domain in yellow. α-helices (cylinders) are shown in a slightly different shade as β-sheets (arrows). α8, α9 and α11 are coloured a darker blue to indicate that they are not part of the $(\alpha/\beta)_8$ barrel. Numbers are given to indicate amino acids at the N-terminus (40) and C-terminus (644+6 – including 6 residues incorporated from the vector) of the structure, as well as the beginning and end of each secondary structure element.

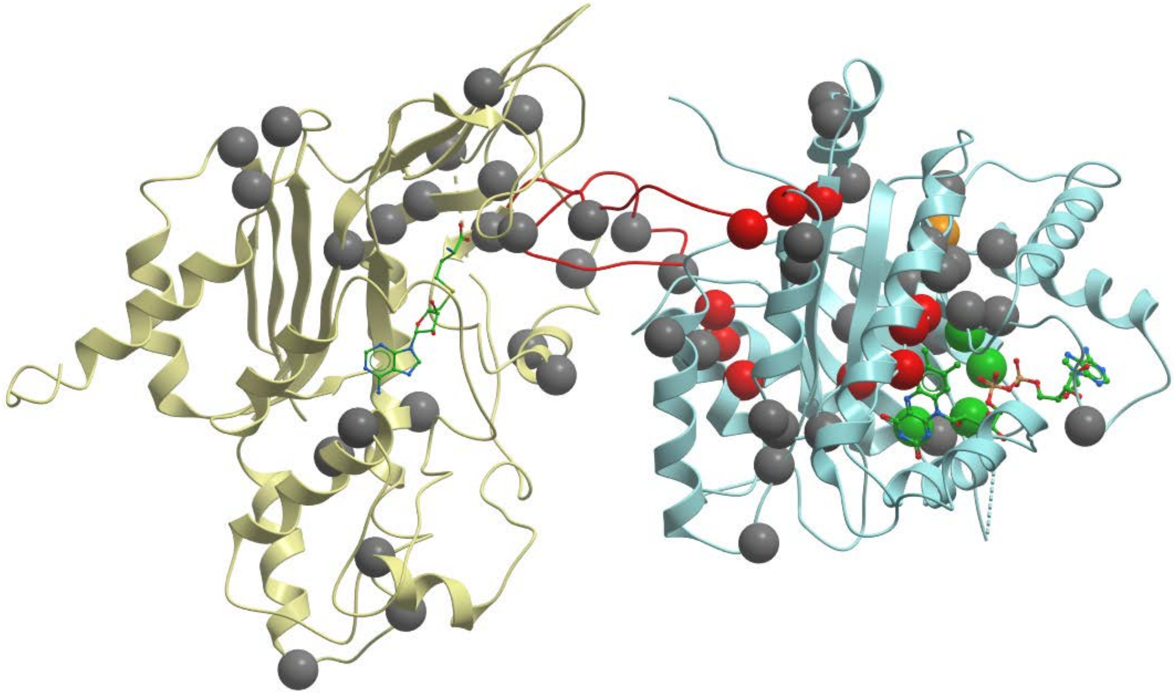

Supplementary Figure 7. Structural analysis of MTHFR mutations. The position of all known disease causing missense mutations represented as spheres mapped onto the *Hs*MTHFR$_{38\text{-}644}$ structure. Green spheres represent residues involved in FAD-binding, red spheres represent mutations that result in massively decreased enzymatic activity, as defined by [1], and grey spheres represent all other mutations. The orange sphere indicates the position of Ala222 found in the common variation Ala222Val.

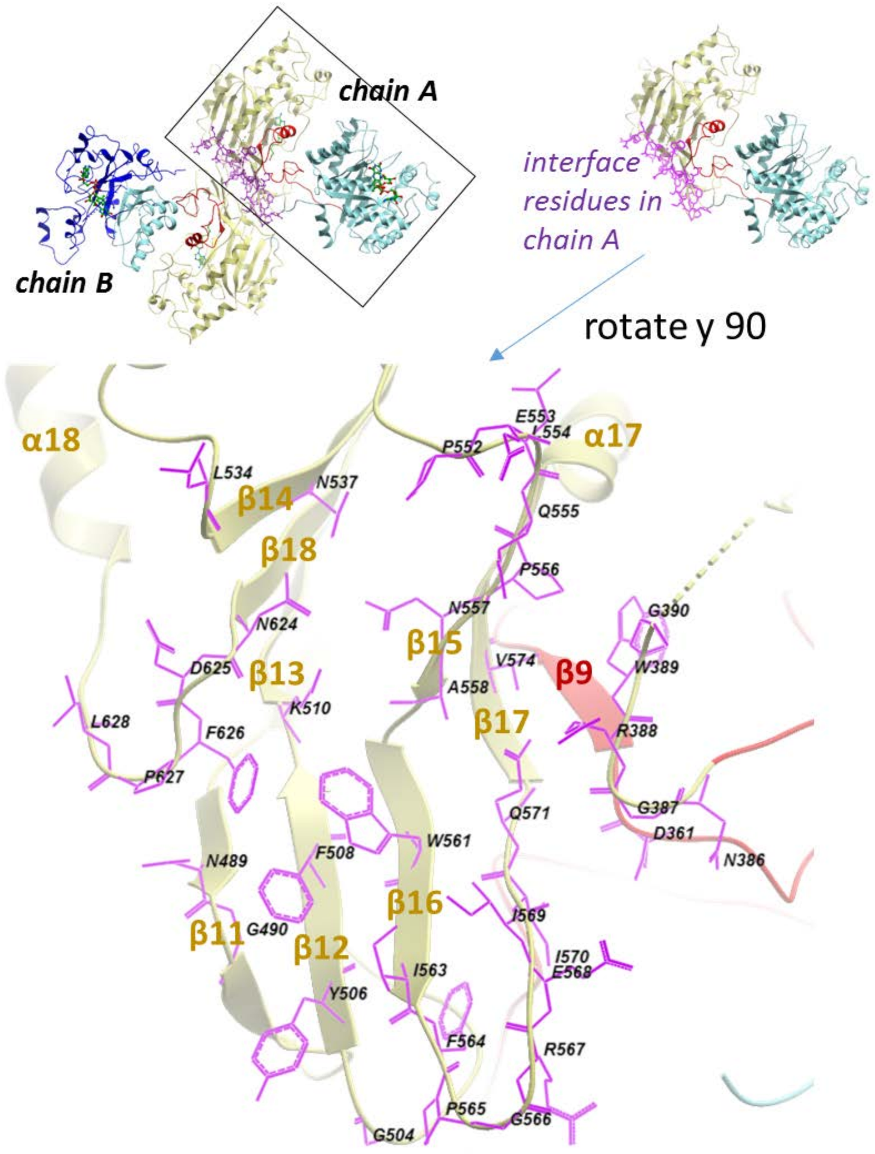

Supplementary Figure 8. Residues contributing to the dimeric interface in the *Hs*MTHFR$_{38\text{-}644}$ crystal structure. Close-up view of the dimeric interface and contributing residues (shown in purple sticks). Secondary structure is colored as in Figure 4.

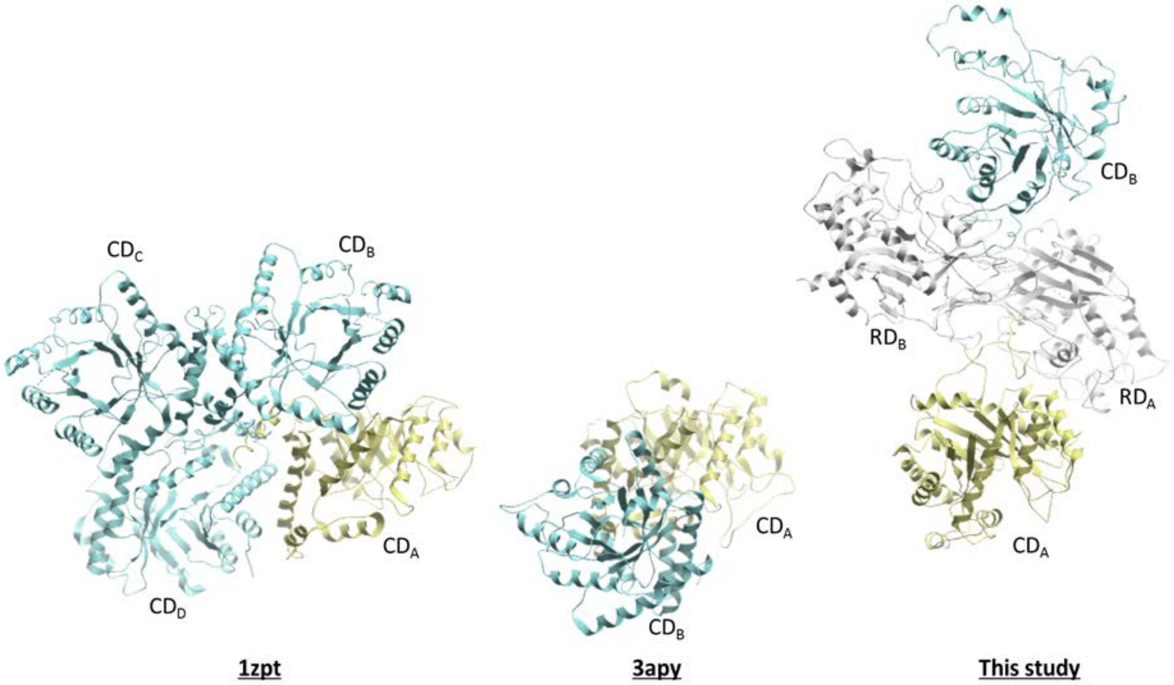

Supplementary Figure 9. Comparison of oligomeric interfaces in MTHFR orthologs. MTHFR from *E. coli* (PDB ID: IZPT) and *Thermus thermophilus* (PDB ID: 3APY) in their higher-order oligomeric states were used to compare against *Hs*MTHFR$_{38\text{-}644}$ (this study). For each, the position of one subunit of the catalytic domain was aligned and kept in a similar orientation (CD_A, shown in yellow), while other catalytic domains are shown in cyan and regulatory domains in grey. *E. coli* MTHFR forms a tetramer of four catalytic subunits. *Thermus thermophilus* forms a dimer, but also only formed of catalytic subunits. By contrast, the catalytic domains do not participate in dimerization of *Hs*MTHFR$_{38\text{-}644}$.

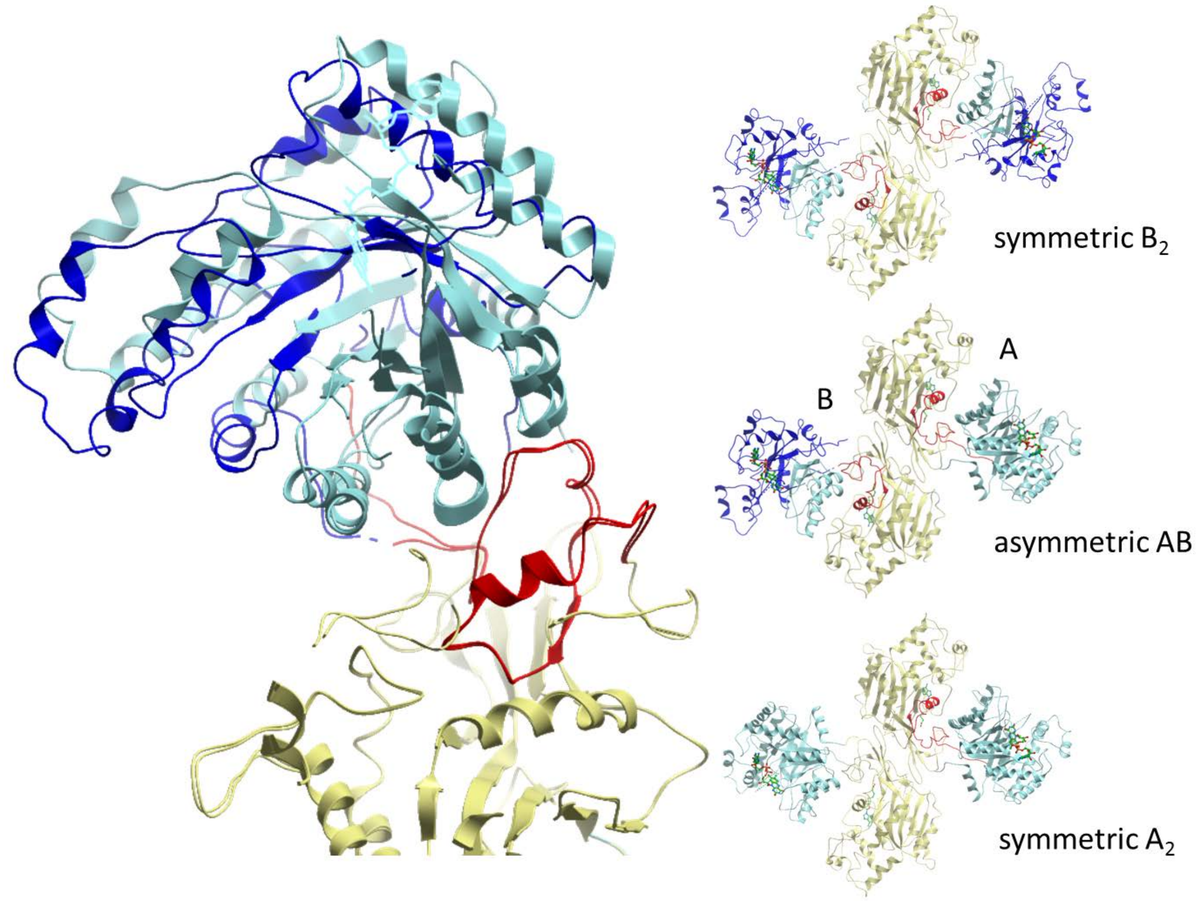

Supplementary Figure 10. Crystal structure of *Hs*MTHFR$_{38\text{-}644}$ depicts an asymmetric dimer. **Main figure:** Overlay and alignment of the regulatory domain (in yellow) to visualize differences in the position of the extended linker region (red) and the ordered (cyan) and disordered (dark blue) catalytic domain of subunits A and B found in the *Hs*MTHFR$_{38\text{-}644}$ structure. FAD is shown in sticks. **Side figures:** Example of the theoretical structures of a symmetrical dimer composed only of subunit B (top) or subunit A (bottom) compared with the experimentally found asymmetric dimer (middle).

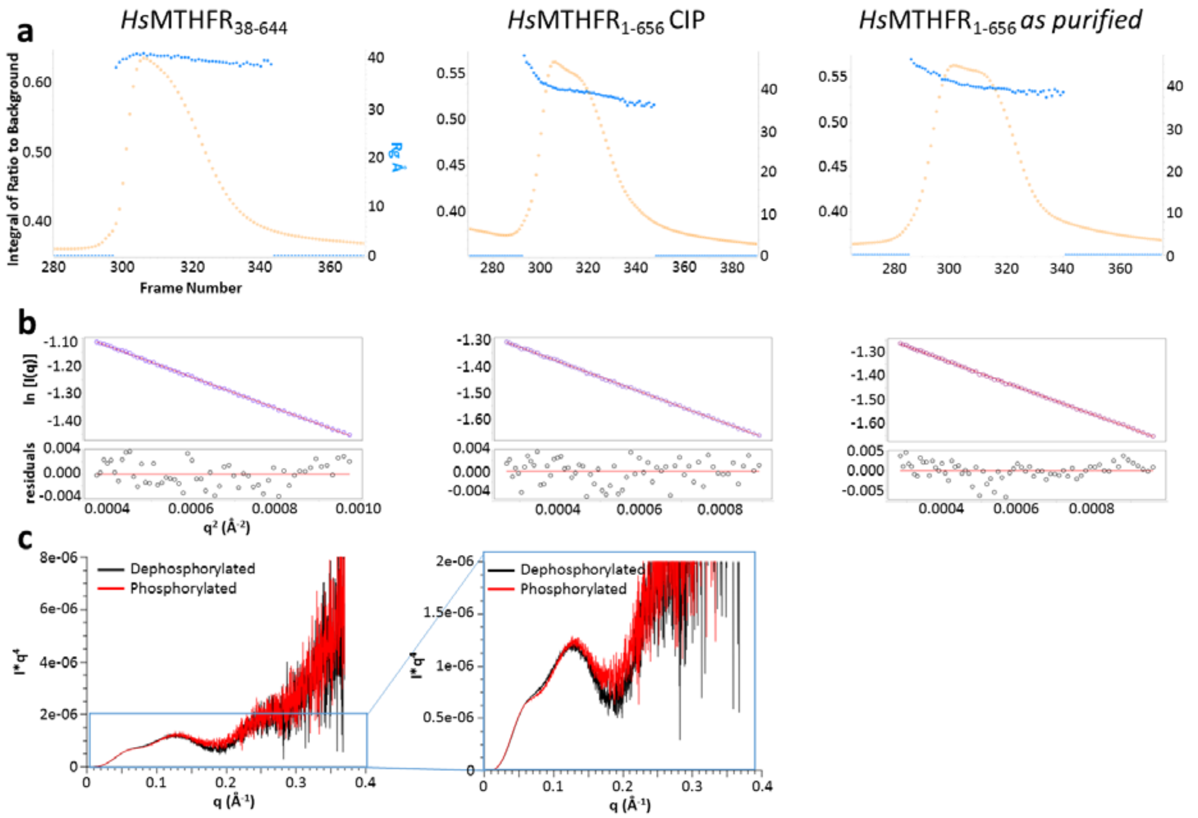

Supplementary Figure 11. SAXS analysis of *Hs*MTHFR. a Analysis of the radius of gyration (Rg) over elution peak in SEC-SAXS. The Rg (blue spheres) and Integral of Ratio to Background (yellow spheres) are plotted versus recorded frames in SEC-SAXS profiles for either *Hs*MTHFR$_{38\text{-}644}$, *Hs*MTHFR$_{1\text{-}656}$ CIP-treated or *Hs*MTHFR$_{1\text{-}656}$ as purified. The frames used for further analysis are, respectively: 305-315, 305-320 and 300-315. Exposure of 3 seconds per frame. **b** Rg (Å) values derived from Guinier are: 40.9 for *Hs*MTHFR$_{38\text{-}644}$, 40.8 for *Hs*MTHFR$_{1\text{-}656}$ CIP-treated and 41.3 for *Hs*MTHFR$_{1\text{-}656}$ as purified. **c** Porod plots of the dephosphorylated/CIP-treated (black) and phosphorylated/as purified (red) data reveal differences in the region around q=0.1 Å^{-1} and the region around q=0.2 Å^{-1} .

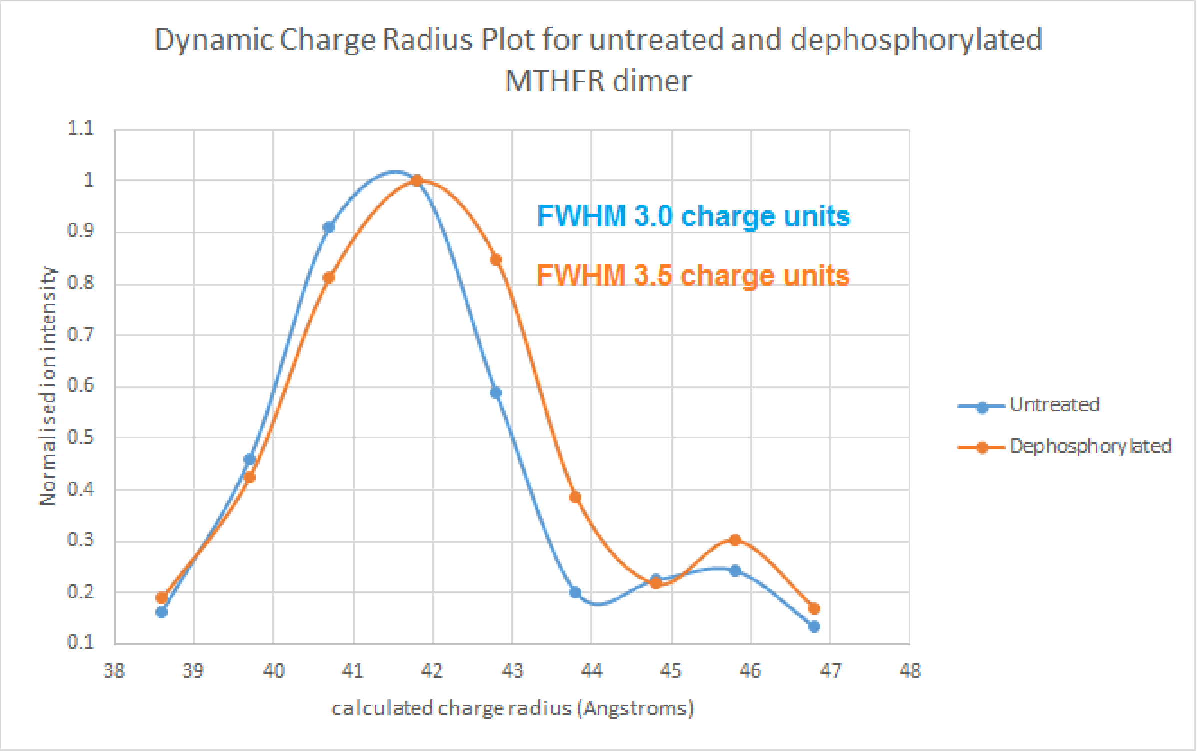

Supplementary Figure 12. Dynamic charge radius plot for *Hs*MTHF$_{1\text{-}656}$. Dynamic charge radius plot of untreated and CIP treated *Hs*MTHFR$_{1\text{-}656}$. The mean calculated charge radii for phosphorylated and dephosphorylated MTHFR at the modal charge state +27 are similar at 41.8 Å. However, dephosphorylation generates a shift in the charge envelope towards higher charge. Comparison of the peak widths (full width at half maximum, FWHM) suggests that dephosphorylated MTHFR is significantly more flexible than the phosphorylated form.

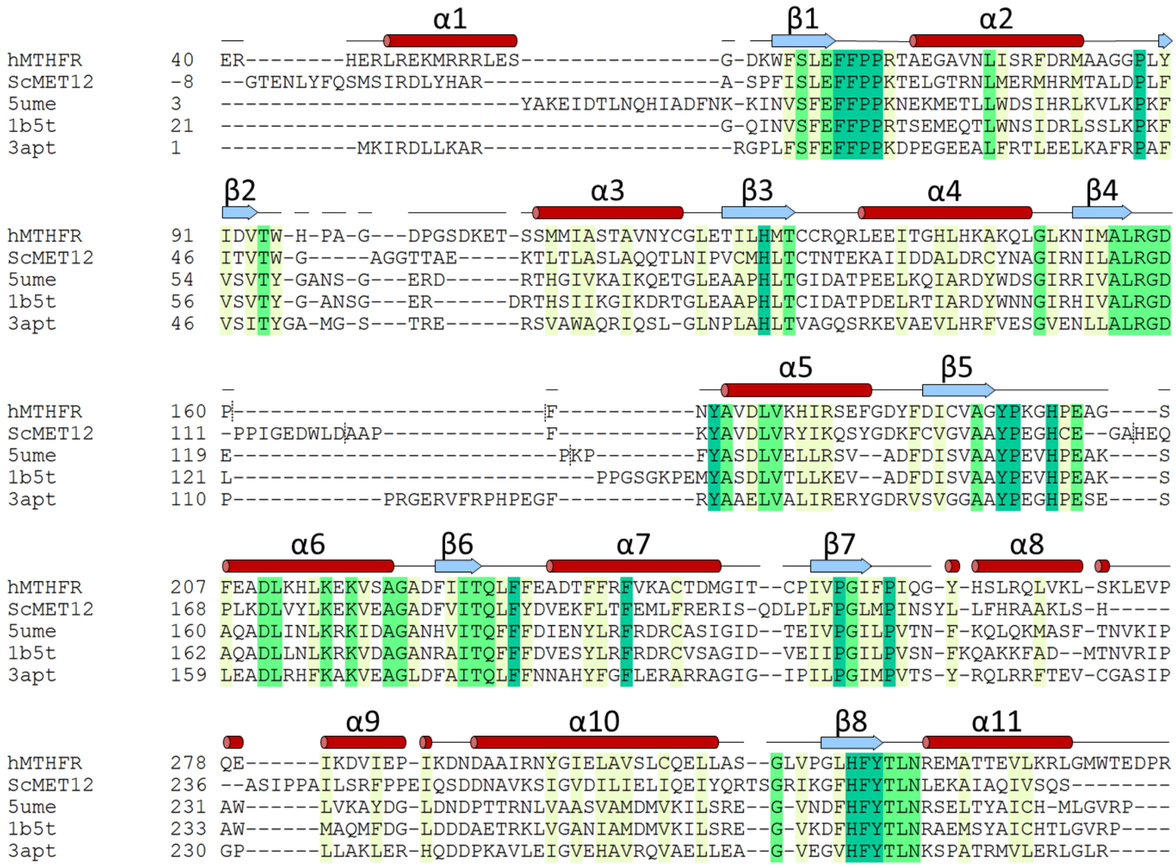

Supplementary Figure 13. Structure-based sequence alignment of the catalytic domain of *Hs*MTHFR$_{38\text{-}644}$ with the catalytic domain of *Sc*MET12 and MetF of Haemophilus influenza (5UME), *Ec*MTHFR (1B5T) and *Tt*MTHFR (3APT). Amino acid conservation is indicated by a coloured background ranging from yellow (conserved in three proteins) to green (invariant residue). Above the alignment the secondary structure of *Hs*MTHFR is given, with red cylinders indicating α-helices and cyan arrows indicating β-sheets.

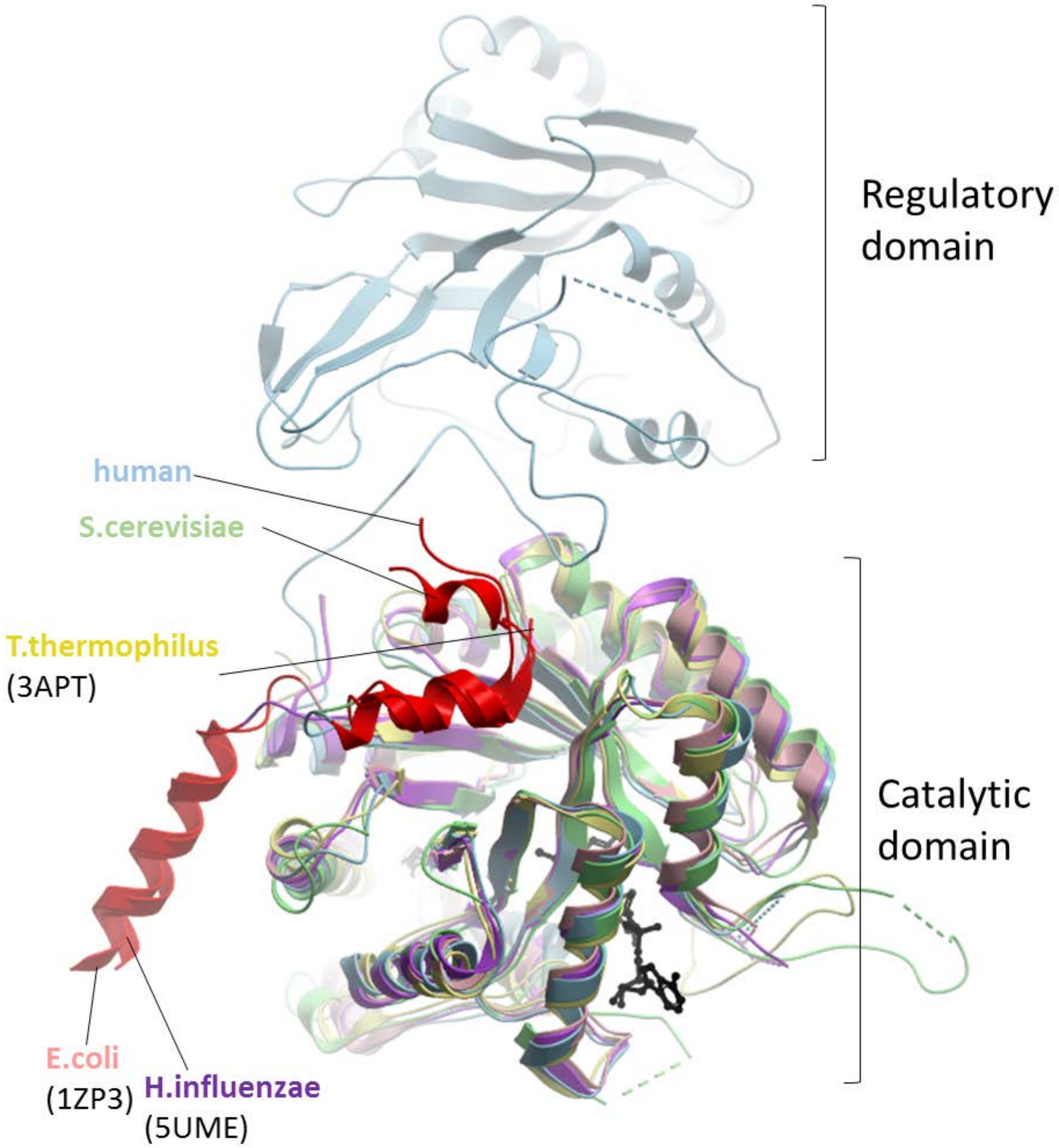

Supplementary Figure 14. Comparison of the N-termini of various MTHFR catalytic domain structures. They include *Hs*MTHFR (blue) and *Sc*MET12 (green) from this study, as well as MTHFR from *T. thermophilus* (yellow), *E. coli* (pink), and *H. influenzae* (purple). In all five structures, the first helix of the catalytic domain α1 is coloured red, to highlight the two different orientations observed.

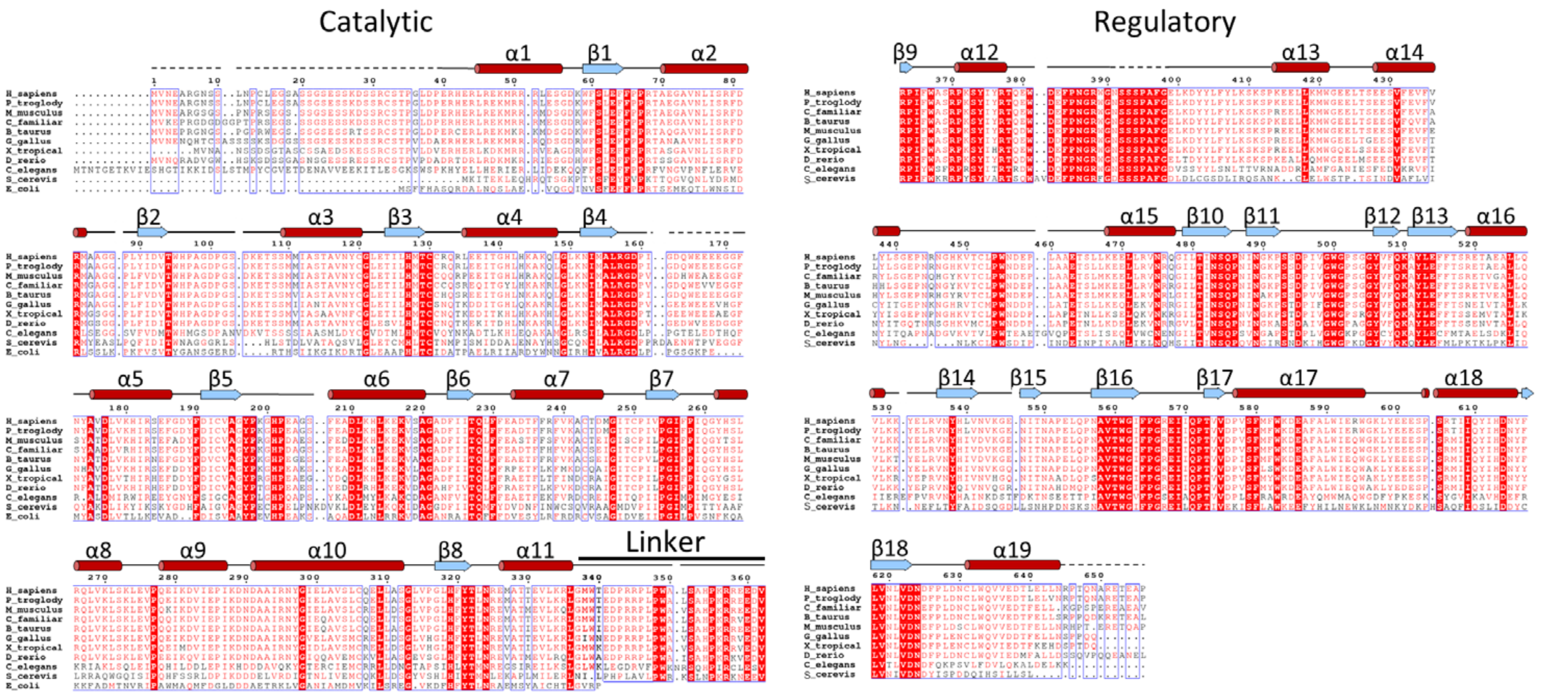

Supplementary Figure 15. Multiple sequence alignment of MTHFR. Multiple sequence alignment was performed for *Homo sapiens* (H_sapiens, NP_005948.3), *Pan troglodytes* (P_troglody, XP_00944660.1), *Mus musculus* (M_musculus, NP_034970.2), *Canus familiarus* (C_familiar, XP_005618050.1), *Bos taurus* (B_taurus, NP_001011685.1), *Gallus* (G_gallus, XP_417645.3), *Xenopus tropicalis* (X_tropical, NP_001096464.1), *Danio rerio* (D_rerio, NP_001268769.1), *Caenorhabditis elegans* (C_elegans, NP_741027.1), *Saccharomyces cerevisiae* (S_cerevis, NP_011390.2), and *Escherichia coli* (E_coli, AJ086043.1) for the catalytic domain, and all but *E. coli* for the linker and regulatory domains. Absolutely conserved residues are highlighted in red shading, well-conserved residues in red font. Secondary structure elements from *Hs*MTHFR$_{38\text{-}644}$ are indicated at the top of the alignment, with red cylinders indicating α-helices and cyan arrows indicating β-sheets. Alignment was created by multalin[2] and visualized by ESPript[3].

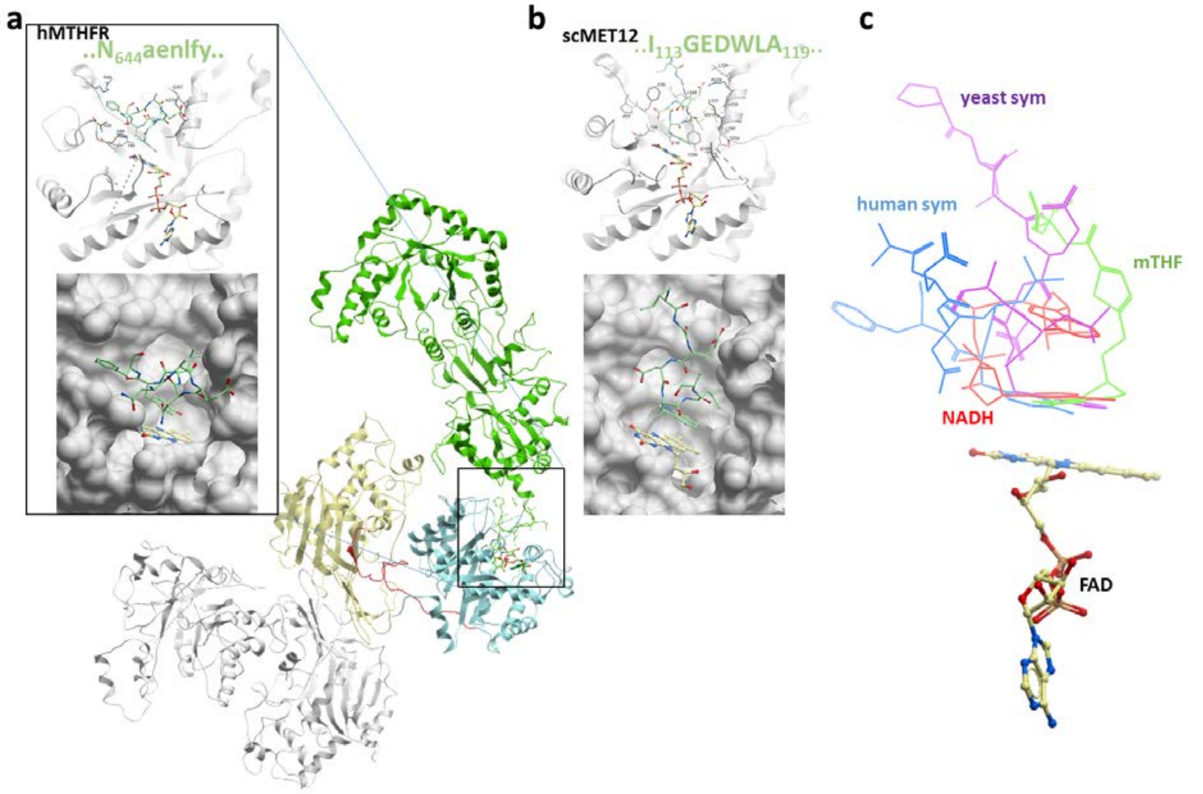

Supplementary Figure 16. Stacking interactions from adjacent crystallographic subunits stabilize the catalytic domains of *Sc*MET12 and *Hs*MTHFR structures. a In *Hs*MTHFR$_{38\text{-}644}$, the final phenylalanine of the uncleaved section of the C-terminal tail His-tag of one subunit stacks against the FAD of chain A of the dimer. The catalytic domain in chain A is therefore held in place by such crystal-mediated packing, resulting in an ordered catalytic domain. This also explains why the catalytic domain in chain B of the dimer, which did not accommodate packing of neighbouring molecule, was largely disordered in the crystal. The additional amino acids contributed by the adjacent unit are shown in green sticks. **b** In the MET12$_{1\text{-}301}$ structure, an extension of the loop p.R$_{108}$-Y$_{129}$ performs this function, whereby p.Trp117 from one-subunit makes stacking interactions with (*si* face of isoalloxazine ring) the FAD from an adjacent subunit. Once again, additional contributing amino acids are depicted in green sticks. **c** Stacking interactions from adjacent subunits in *Hs*MTFHR (blue) and *Sc*MET12 (purple) extend into the active site to interact with FAD, and thus overlap with the NAD(P)H (red) and CH_3-THF (green) binding sites.

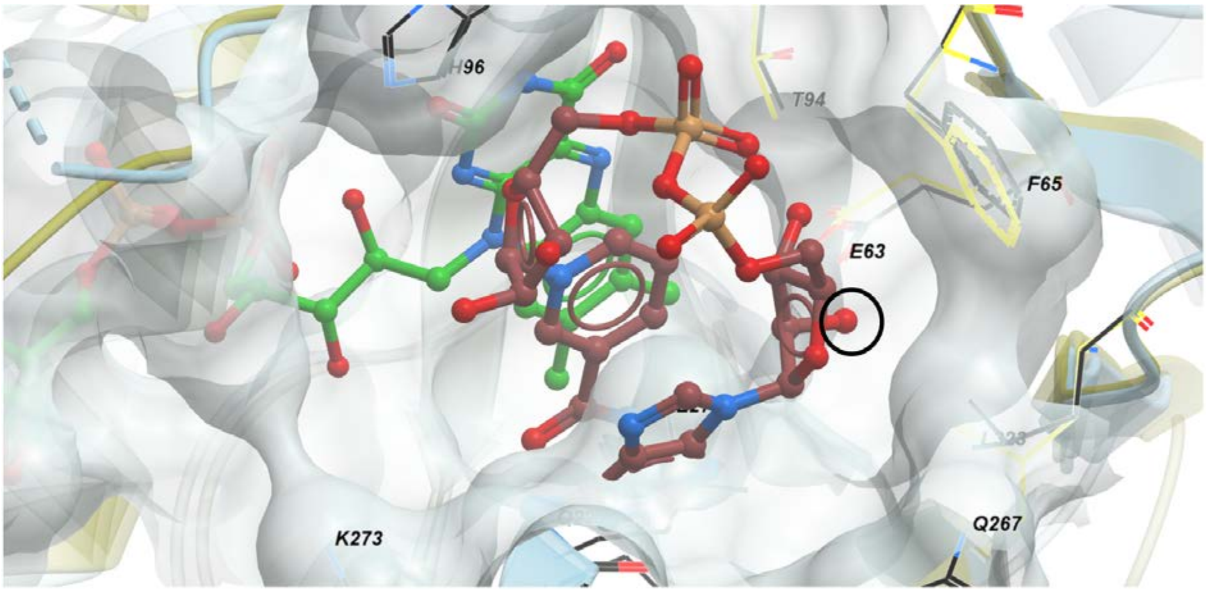

Supplementary Figure 17. Close-up view of the NADH binding pocket indicating lack of specific accommodation for an additional ribosyl phosphate. Structural overlay of *Hs*MTHFR$_{38\text{-}644}$ (cyan) and *Ec*MTHFR (PDB: IZRQ, yellow) with residues contributing the binding pocket shown in sticks (*Hs*MTHFR: black; *Ec*MTHFR: yellow) and labeled according to *Hs*MTHFR. The ribosyl oxygen which accommodates the additional phosphate group in NADPH is circled in black. Notice the strong sequence and structural conservation of amino acids in the area surrounding this ribosyl oxygen.

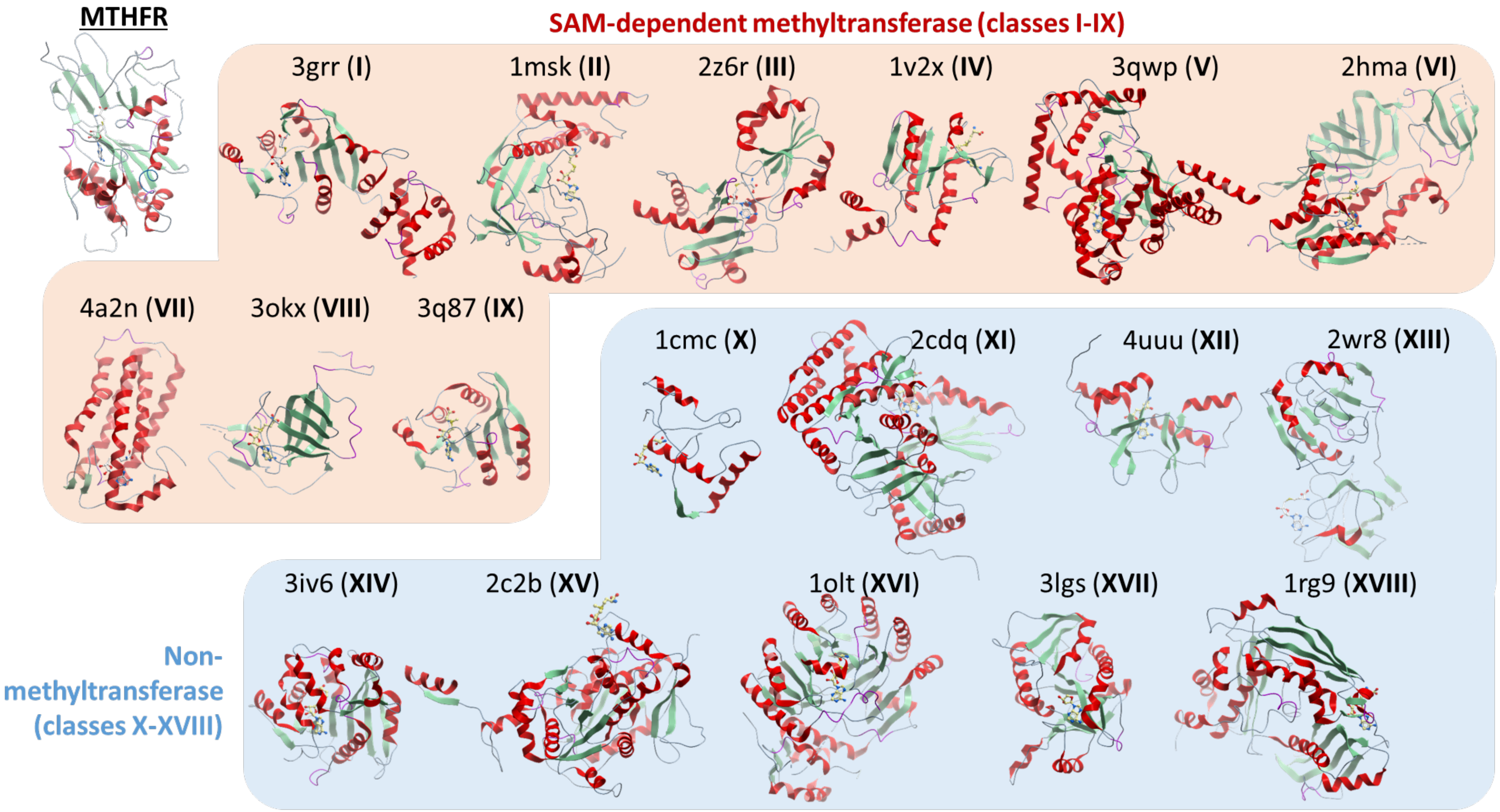

Supplementary Figure 18. Cartoon representation of SAM binding domain of *Hs*MTHFR and other known SAM-binding domains. The *Hs*MTHFR regulatory domain fold is displayed alongside with representative examples from 18 known SAM-binding folds from methyltransferases (classes I-IX) and non-methyltransferases (classes X-XVIII). All the structures are shown with the SAM ligand (sticks) in equivalent orientation.

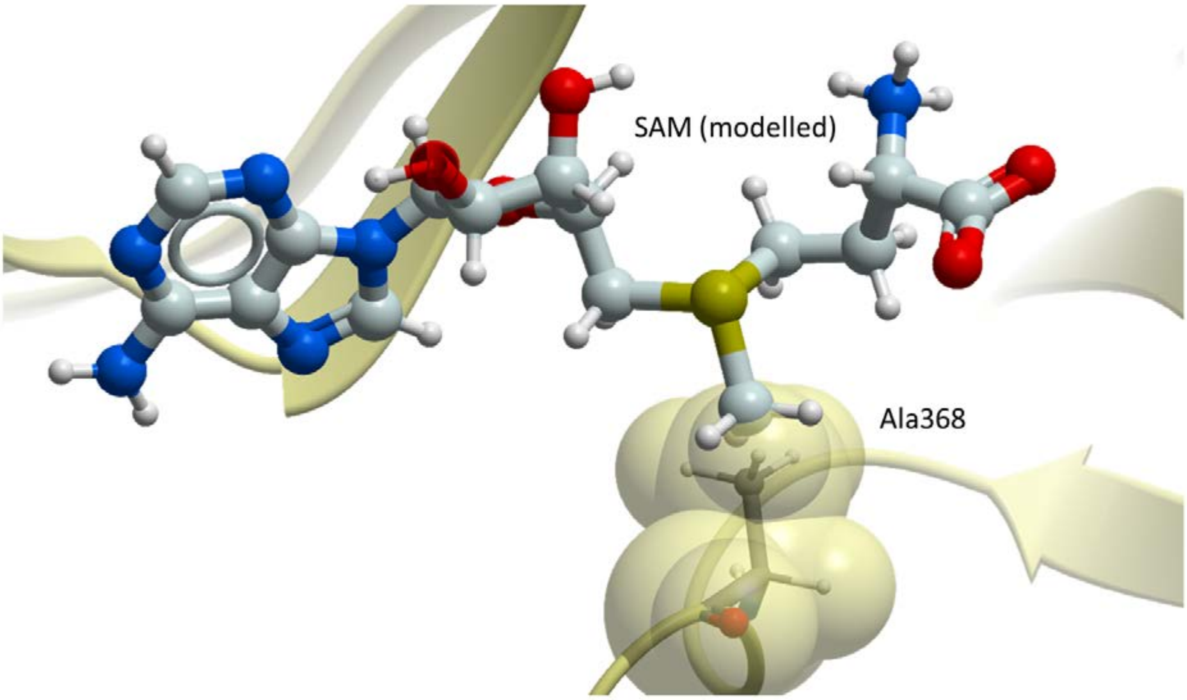

Supplementary Figure 19. Clash between modelled SAM and Ala368 of *Hs*MTHFR$_{38\text{-}644}$.

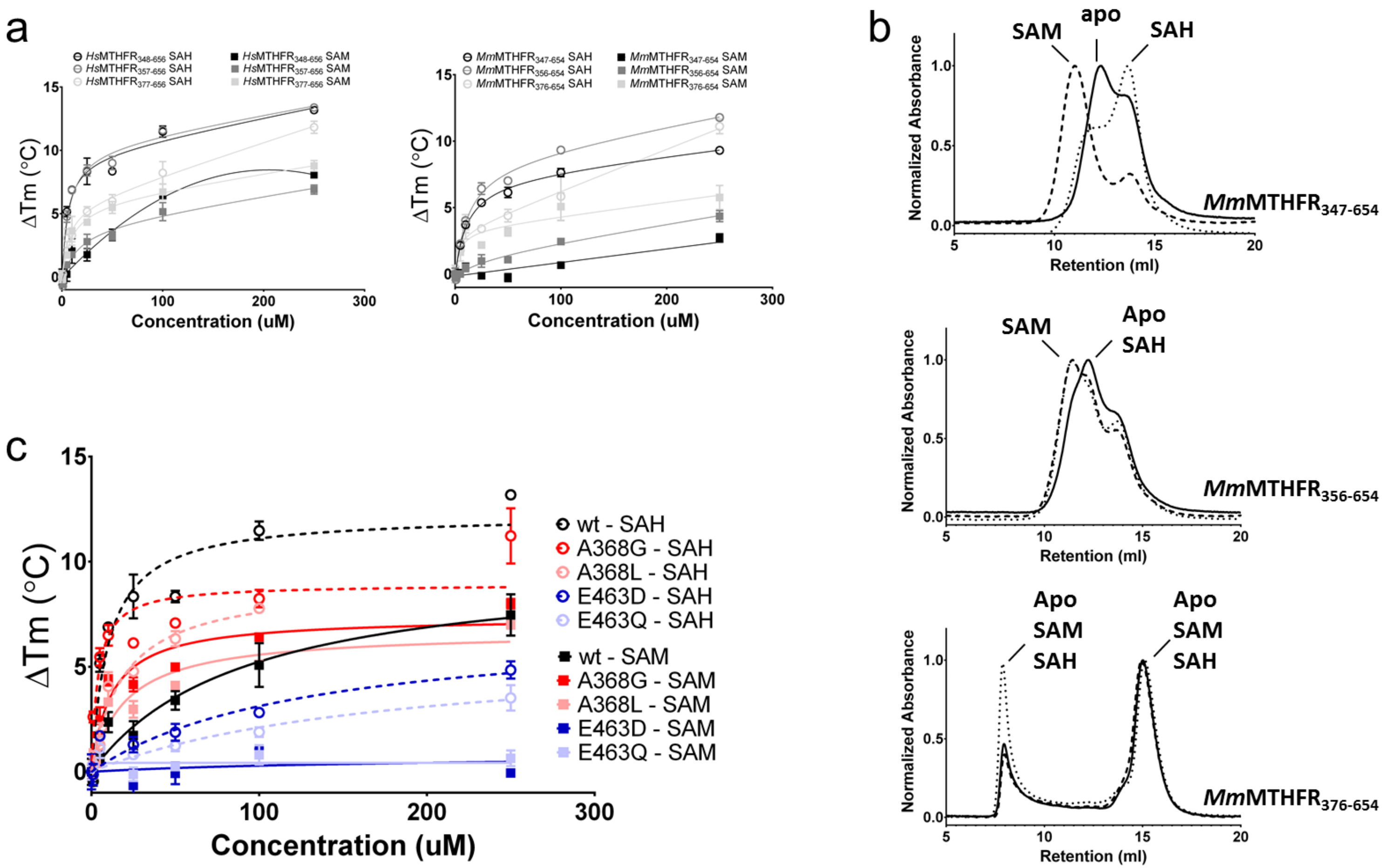

Supplementary Figure 20. SAH/SAM binding and conformational change in N-terminally truncated *Hs*MTHFR and *Mm*MTHFR. a Differential scanning fluorimetry of N-terminally truncated *Hs*MTHFR (left panel) and mouse (*Mm*)MTHFR (right panel) following incubation with increasing concentrations of SAH or SAM. Lines represent best fit curves of one-site binding calculated by GraphPad v6.0. **b** Size exclusion chromatography of *Mm*MTHFR with various N-terminal truncations following incubation with SAM (dashed lines), SAH (dotted lines), or nothing (apo; solid line). **c** Differential scanning fluorimetry of *Hs*MTHFR$_{348\text{-}656}$ protein without (wt) or carrying the corresponding mutations following incubation with increasing concentrations of SAH or SAM. Lines represent best fit curves of one-site binding of SAH (dashed) or SAM (solid) as calculated by GraphPad v6.0. For **a** and **c**, each value represents the results of at least 3 separate experiments and is given as ± S.D.

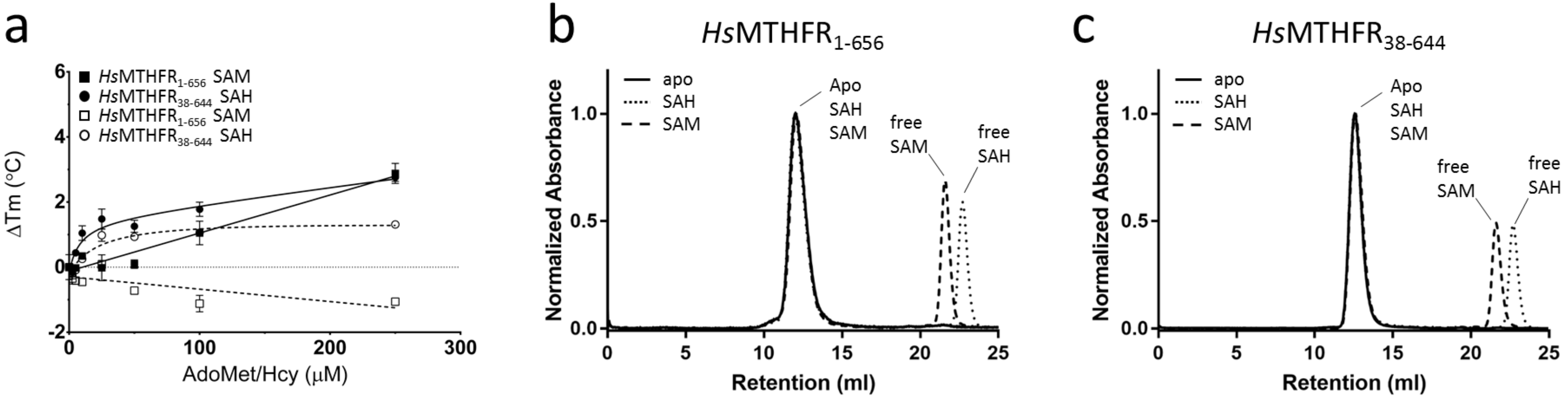

Supplementary Figure 21. SAH/SAM binding does not induce conformational change in *Hs*MTHFR$_{1\text{-}656}$ or *Hs*MTHFR$_{38\text{-}644}$. a Differential scanning fluorimetry of *Hs*MTHFR following incubation with increasing concentrations of SAH or SAM. Each value represents the results of at least 3 separate experiments and is given as ± S.D. Lines represent best fit curves of one-site binding calculated by GraphPad v6.0. **b** Size exclusion chromatography of *Hs*MTHFR$_{1\text{-}656}$ following incubation with SAM (dashed lines), SAH (dotted lines), or nothing (apo; solid line). **c** Size exclusion chromatography of *Hs*MTHFR$_{38\text{-}644}$ following incubation with SAM (dashed lines), SAH (dotted lines), or nothing (apo; solid line).

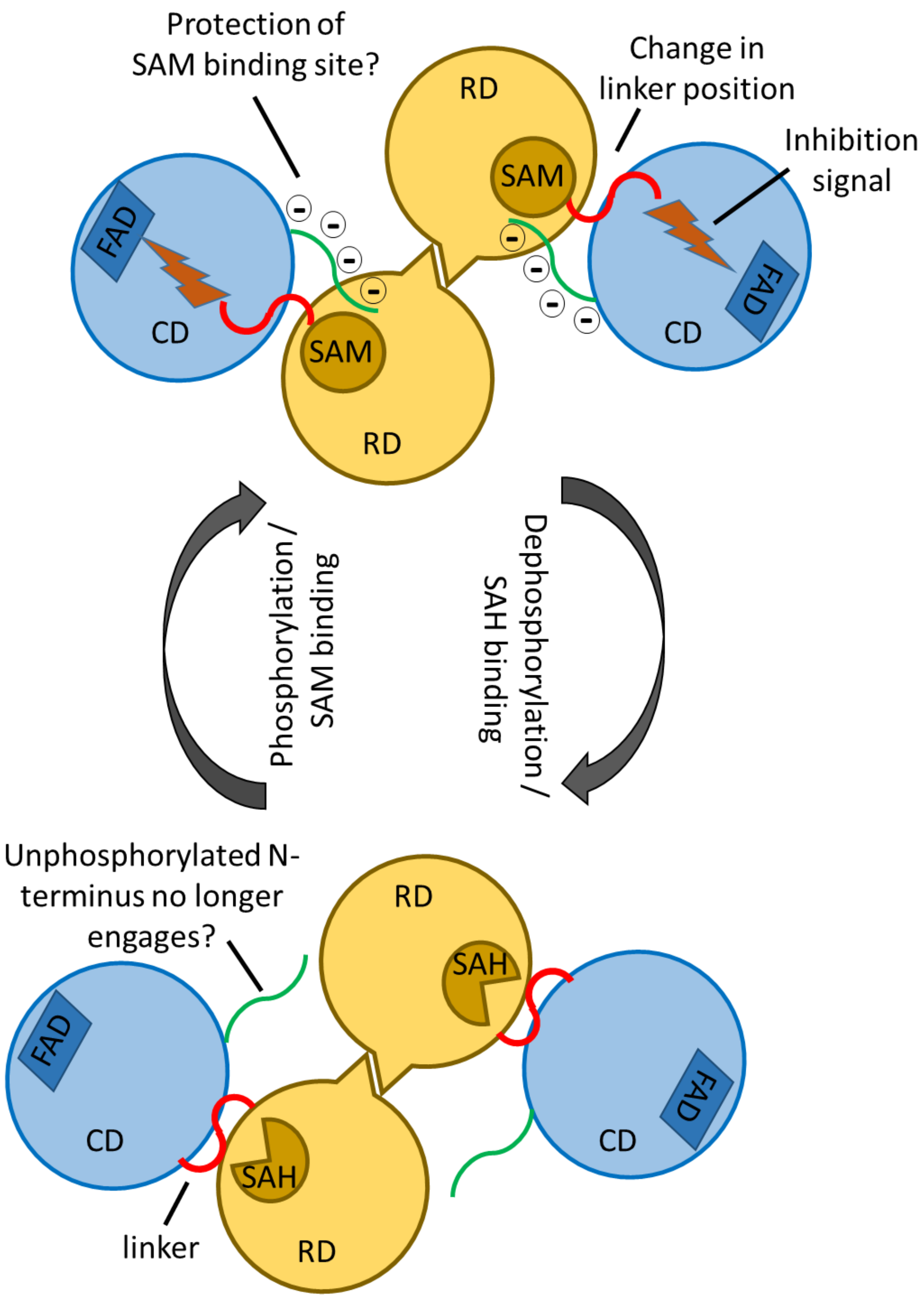

Supplementary Figure 22. Schematic mechanism of human MTHFR postulated from this study. N-terminal phosphorylation region is coloured green, catalytic domain (CD) brown, regulatory domain (RD) blue, and inter-domain linker red.

Supplementary Table 1. SAXS analysis of *Hs*MTHFR

	MTHFR $_{1\text{-}656}$ CIP-treated	MTHFR $_{1\text{-}656}$ as-purified
**Structural parameters**		
Rg (Å , from Guinier)	40.8 ± 0.5	41.3 ± 0.5
Volume (Å^3, from Porod)	220	254
**Mr determination (kDa)[1]**		
Estimated from Porod volume	137	158
Estimated from volume-of-correlation (Vc)	140	150
**Fit to CORAL model generated from *Hs*MTHFR$_{38\text{-}644}$[2]**		
Chi-square	23.2 ± 1.2	29.8 ± 1.6
**Fit to scattering back-calculated from *Hs*MTHFR$_{38\text{-}644}$ structure[3]**		
Chi-square	5.9	11.6

[1]Mr calculated from sequence of a *Hs*MTHFR$_{38\text{-}644}$ dimer: 150.7 kDa

[2]To clarify the difference in overall shape, we fitted the theoretical scattering curves of the *Hs*MTHFR$_{38\text{-}644}$ rigid body CORAL models to the SAXS experimental data of dephosphorylated and phosphorylated MTHFR.

[3]We also fit the theoretical scattering curve of *Hs*MTHFR$_{38\text{-}644}$ observed in the crystal, to the experimental data of dephosphorylated and phosphorylated MTHFR

Supplementary References

1. Burda P, *et al.* Insights into severe 5,10-methylenetetrahydrofolate reductase deficiency: molecular genetic and enzymatic characterization of 76 patients. *Hum Mutat* **36**, 611-621 (2015).

2. Corpet F. Multiple sequence alignment with hierarchical clustering. *Nucleic Acids Res* **16**, 10881-10890 (1988).

3. Gouet P, Robert X, Courcelle E. ESPript/ENDscript: Extracting and rendering sequence and 3D information from atomic structures of proteins. *Nucleic Acids Res* **31**, 3320-3323 (2003).
